# Transferrin-Conjugated, Camptothecin-Bearing Dendrimersomes Entrapping Docetaxel as a Dual-Drug Nanoplatform for Targeted Prostate Cancer Therapy

**DOI:** 10.3390/pharmaceutics18080959

**Published:** 2026-08-04

**Authors:** Musa Albatsh, Zainab Al-Quraishi, Partha Laskar, Sukrut Somani, Craig Irving, Graeme R. Mackenzie, Stuart Woods, Craig W. Roberts, Margaret Mullin, Christine Dufès

**Affiliations:** 1 Strathclyde Institute of Pharmacy and Biomedical Sciences, University of Strathclyde, 161 Cathedral Street, Glasgow G4 0RE, UK; malbatsh@meu.edu.jo (M.A.); zainab.alsiryawi.2021@uni.strath.ac.uk (Z.A.-Q.); plaskar@gitam.edu (P.L.); sukrut.somani@outlook.com (S.S.); graeme.mackenzie@strath.ac.uk (G.R.M.); stuart.woods@uws.ac.uk (S.W.); c.w.roberts@strath.ac.uk (C.W.R.); 2 Faculty of Pharmacy, Middle East University, Queen Aliaa Airport Street, Amman 11610, Jordan; 3 Department of Chemistry, School of Science, Gandhi Institute of Technology and Management, Visakhapatnam 530045, India; 4 Department of Pure and Applied Chemistry, University of Strathclyde, 295 Cathedral Street, Glasgow G1 1XL, UK; craig.irving@strath.ac.uk; 5 School of Health and Life Sciences, University of the West of Scotland, Paisley Campus, High Street, Paisley PA1 2BE, UK; 6 Cell Analysis Facility, Medical and Veterinary & Life Sciences Shared Research Facilities, College of Medical, Veterinary and Life Sciences, University of Glasgow, Glasgow G12 8QQ, UK; margaret.mullin@glasgow.ac.uk

**Keywords:** dendrimer, dendrimersome, camptothecin, docetaxel, transferrin, prostate cancer, cellular uptake, cancer therapy

## Abstract

**Background/Objectives**: Advanced prostate cancer remains difficult to treat because docetaxel, although clinically important, is limited by systemic toxicity, poor tumor selectivity, and acquired resistance. Camptothecin is a potent anticancer agent, but its clinical application is restricted by poor solubility and instability. This study investigated the synergy between docetaxel and camptothecin and developed transferrin-conjugated, camptothecin-bearing dendrimersomes entrapping docetaxel as a targeted nanocarrier for prostate cancer therapy. **Methods:** Drug synergy was evaluated in PC3-Luc cells using an MTT assay and combination index analysis. Transferrin-conjugated, disulfide-linked camptothecin-bearing PEGylated DAB dendrimers were synthesized and characterized by ^1^H-NMR, critical aggregation concentration analysis, transmission electron microscopy, and entrapment efficiency measurements. pH- and redox-dependent drug release was assessed by dialysis. Cellular uptake and uptake mechanisms were investigated by confocal microscopy, flow cytometry, and inhibitor studies in PC3-Luc, DU145, and LNCaP cells. Anti-proliferative efficacy was determined by an MTT assay. **Results**: Docetaxel and camptothecin showed marked synergy in PC3-Luc cells, with a minimum combination index of 0.20 ± 0.01 and 88.10 ± 0.41% growth inhibition at low nanomolar concentrations. Transferrin-conjugated dendrimersomes self-assembled into spherical vesicles with a critical aggregation concentration of approximately 250 µg/mL. They had high docetaxel entrapment efficiency (89.10 ± 0.08%) and enhanced drug release under acidic and reductive conditions. They significantly increased cellular uptake of docetaxel relative to non-targeted dendrimersomes (by up to 3-fold) and free drugs (by up to 20-fold), mainly through transferrin receptor-mediated endocytosis, and improved anti-proliferative activity in all three cell lines. Tf-conjugated DPSSC produced the lowest IC_50_ values among the tested formulations: 11.72 ± 1.02 nM in PC3-Luc, 9.88 ± 1.22 nM in DU145, and 7.88 ± 1.35 nM in LNCaP cells. **Conclusions**: Transferrin-conjugated camptothecin-based dendrimersomes entrapping docetaxel represent a promising multifunctional nanocarrier for prostate cancer that combines synergistic dual-drug therapy, active targeting, and stimulus-responsive release, supporting further evaluation as a selective delivery strategy in advanced prostate cancer using preclinical models and in vivo studies.

## 1. Introduction

Prostate cancer is one of the most frequently diagnosed malignancies in men worldwide and remains a leading cause of cancer-related mortality, with recent global analyses confirming a substantial and geographically heterogeneous disease burden [1,2]. Although localized disease can often be managed by surgery and radiotherapy, advanced, metastatic, and castration-resistant prostate cancer still requires systemic treatment and continues to represent a major clinical challenge [1,2].

Docetaxel (DTX) remains a cornerstone of chemotherapy for advanced prostate cancer, including metastatic castration-resistant prostate cancer. As a taxane, it promotes tubulin polymerization and stabilizes microtubules, thereby disrupting mitosis and inhibiting tumor cell proliferation. However, its clinical benefit is frequently limited by poor aqueous solubility, systemic toxicity, and the emergence of resistance. Several mechanisms have been implicated in docetaxel resistance, including increased drug efflux, alterations in microtubule dynamics, changes in androgen receptor signaling, epithelial–mesenchymal transition, and dysregulation of apoptotic pathways. Cabazitaxel was subsequently introduced for patients progressing after docetaxel therapy, but therapeutic outcomes remain suboptimal for many patients, highlighting the continued need for more effective and selective treatment strategies [3,4,5,6].

Camptothecin (CPT), a potent topoisomerase I inhibitor, is another anticancer agent with promising activity against a broad range of malignancies, including prostate cancer. Its therapeutic application, however, has been restricted by major pharmaceutical limitations, notably poor aqueous solubility, instability of the active lactone ring at physiological pH, rapid systemic clearance, and dose-limiting toxicity [7,8,9]. One attractive strategy to overcome these limitations is the use of rational drug combinations. Combining agents with distinct and complementary mechanisms of action can enhance anti-proliferative efficacy, reduce the likelihood of resistance, and lower the dose required for each individual drug [10]. In parallel, nanocarrier-based co-delivery systems can help maintain synergistic drug ratios and improve tumor-directed delivery. Recent studies have demonstrated that co-delivery of docetaxel with doxorubicin, resveratrol, or brusatol can enhance antitumor activity in prostate cancer models, including in PSMA-targeted formulations [11,12,13,14]. However, a transferrin-targeted system co-delivering docetaxel and camptothecin has not previously been reported.

Among nanocarrier platforms, dendrimers have attracted considerable interest because of their highly branched architecture, internal cavities, multivalency, and ease of chemical modification. Diaminobutyric polypropylenimine (DAB) dendrimers are particularly attractive for drug and gene delivery because of their strong interaction with therapeutic cargoes and their capacity for functional surface engineering. PEGylation further improves their colloidal behavior, biocompatibility, and circulation-related properties, while also providing reactive groups for ligand conjugation. Importantly, previous work from our group has shown that PEGylated DAB dendrimers can provide efficient delivery while reducing cytotoxicity, and that hydrophobically modified DAB dendrimers can self-assemble into dendrimersomes suitable for drug and gene delivery. In particular, redox-sensitive cholesterol-bearing and camptothecin-bearing PEGylated DAB dendrimers have been shown to form dendrimersomes with stimulus-responsive behavior, supporting their potential as multifunctional nanocarriers for cancer therapy [15,16,17].

Active targeting represents a further strategy to improve the therapeutic selectivity of nanocarriers. Among the targeting ligands explored in cancer nanomedicine, transferrin (Tf) is particularly attractive because its receptor, transferrin receptor 1 (TfR1), is overexpressed in many malignant cells, including prostate cancer cells, reflecting their increased iron requirements and altered iron metabolism. Binding of Tf-functionalized nanocarriers to TfR1 promotes receptor-mediated endocytosis and can enhance intracellular delivery of therapeutic cargoes. Tf/TfR-targeted delivery has been widely investigated in cancer, and our group has contributed substantially to this field through work on transferrin receptor-targeted delivery systems and transferrin-bearing DAB dendrimers. In prostate cancer specifically, transferrin-conjugated dendriplexes from our laboratory have previously shown therapeutic efficacy after intravenous administration, while more recent work from the group demonstrated the utility of transferrin-bearing, zein-based hybrid lipid nanoparticles for drug and gene delivery to prostate cancer cells. These observations, together with earlier reports on transferrin-targeted taxane nanocarriers, provide a strong rationale for using Tf as a targeting ligand in the present work [18,19,20,21,22,23,24,25,26]. The recent literature has further emphasized both the versatility and the limitations of TfR1-directed delivery, including the influence of receptor distribution, ligand density, endogenous transferrin competition, and off-target biodistribution [27,28,29,30].

In this study, we first investigated the synergistic interaction between docetaxel and camptothecin in PC3-Luc prostate cancer cells. Transferrin-conjugated, camptothecin-based dendrimersomes containing docetaxel were then prepared and assessed for physicochemical properties, drug release, cellular internalization, uptake mechanisms, and anti-proliferative activity in PC3-Luc, DU145, and LNCaP prostate cancer cell lines. By integrating synergistic dual-drug treatment, transferrin-mediated active targeting, and stimulus-responsive delivery within a single nanocarrier, this work aims to advance a more effective and selective nanomedicine strategy for prostate cancer therapy. Part of the experimental work underpinning this article was originally undertaken and reported within the doctoral research of Musa Albatsh [31].

## 2. Materials and Methods

### 2.1. Cell Lines and Reagents

Generation 3 diaminobutyric poly(propylenimine) (DAB) dendrimer (with a molecular weight of 1686.79 Da and 16 terminal primary amino groups), human holo-transferrin (Tf), docetaxel (DTX), camptothecin (CPT), glutathione (GSH), 3-tritylsulfanylpropionic acid, 4-dimethylaminopyridine (DMAP), N-(3-dimethylaminopropyl)-N′-ethylcarbodiimide hydrochloride (EDC), Nile red, anhydrous dichloromethane, triethylsilane, trifluoroacetic acid, diethyl ether, deuterated dimethyl sulfoxide-d_6_ (DMSO-d_6_), 2-iminothiolane hydrochloride (Traut’s reagent), deuterated chloroform (CDCl_3_), deuterium oxide, phenylarsine oxide (PhAsO), filipin (Fil), colchicine (Colch) and poly-L-lysine (PLys) were purchased from Sigma-Aldrich (Poole, UK), unless stated otherwise. The cross-linker N-γ-maleimidobutyryl-oxysuccinimide ester (GMBS) was obtained from PolyPeptide (Strasbourg, France). Orthopyridyl disulfide polyethylene glycol (OPSS-PEG) succinimidyl carboxymethyl ester (OPSS-PEG-SCM), which contained PEG with a nominal molecular weight of 2000 Da, was purchased from JenKem Technology (Plano, TX, USA). Bioware^®^ PC-3M-luc-C6 human prostate adenocarcinoma cells expressing firefly luciferase (PC3-Luc; androgen-insensitive) were purchased from Caliper Life Sciences (Hopkinton, MA, USA), while the androgen-insensitive DU145 and androgen-sensitive LNCaP human prostate carcinoma cell lines were obtained from the European Collection of Cell Cultures (Salisbury, UK). Cell culture media were obtained from Life Technologies (Paisley, UK). Vectashield^®^ mounting medium containing propidium iodide was purchased from Vector Laboratories (Peterborough, UK).

### 2.2. Cell Culture

PC3-Luc and DU145 cells were propagated at 37 °C and 5% CO_2_ in humidified incubators using Minimum Essential Medium supplemented with 10% (*v*/*v*) fetal bovine serum, 1% (*v*/*v*) L-glutamine, and 0.5% (*v*/*v*) penicillin–streptomycin. LNCaP cells were maintained under the same atmospheric conditions in RPMI medium containing 10% (*v*/*v*) fetal bovine serum, 1% (*v*/*v*) L-glutamine, sodium pyruvate (100 mM), HEPES solution (1 M), and 0.5% (*v*/*v*) penicillin–streptomycin.

### 2.3. Drug Synergy

PC3-Luc cells were plated at 5000 cells per well in 96-well plates and allowed to grow for 48 h before exposure to docetaxel/camptothecin combinations ranging from 250 to 1.95 nM. Untreated wells served as negative controls, whereas 1% (*w*/*v*) Triton X-100 in PBS provided the positive control. Following 48 h of treatment, dose–response curves were generated to assess the potential synergistic interaction between docetaxel and camptothecin. Growth inhibitory concentrations were determined by plotting the percentage of cell viability against the logarithm of the treatment concentration using OriginPro^®^ 2021 software (OriginLab^®^ Corporation, Northampton, MA, USA). Combination index (CI) values were then calculated using CompuSyn^®^ software (ComboSyn Inc., Paramus, NJ, USA), where CI < 1 indicates synergism, CI = 1 indicates an additive effect, and CI > 1 indicates antagonism [10].

### 2.4. Synthesis of Transferrin-Conjugated, Disulfide-Linked Camptothecin-Bearing PEGylated DAB Dendrimer (Tf-Conjugated DPSSC)

#### 2.4.1. Synthesis of Thiolated Camptothecin

Thiolated camptothecin was synthesized using a method outlined by Zhou et al. [32], as documented in the associated doctoral research [31]. Camptothecin (40 mg, 0.1148 mmol), 3-tritylsulfanylpropionic acid (40 mg, 0.1148 mmol), DMAP (24 mg, 0.1252 mmol), and EDC·HCl (15 mg, 0.1228 mmol) were combined in 4 mL anhydrous dichloromethane and stirred overnight at 20 °C. After filtration through Whatman cellulose paper (18.5 cm diameter; 11 μm pores), the solvent was evaporated overnight. The residue was redissolved in 4 mL anhydrous dichloromethane, followed sequentially by triethylsilane (0.5 mL) and trifluoroacetic acid (0.8 mL), and mixed for 2 h at 20 °C. Volatile solvents were removed overnight. The remaining material was dissolved in 500 μL dichloromethane, precipitated into 15 mL cold diethyl ether, collected, and dried under vacuum.

#### 2.4.2. Synthesis of Disulfide-Linked, Camptothecin-Bearing PEGylated Dendrimer (DAB-PEG-SS-CPT or DPSSC)

The disulfide-linked, camptothecin-bearing PEGylated dendrimer (DAB–PEG–SS–CPT; DPSSC) was synthesized using a method described by Laskar et al. [16]. DAB, OPSS-PEG-SCM and thiolated CPT were introduced at a nominal molar feed ratio of 1:1:2, respectively. The same DPSSC construct prepared using this synthetic procedure was previously characterized by MALDI-TOF mass spectrometry and had an average molecular weight of approximately 4480 Da, consistent with average PEG: DAB and CPT: DAB substitution ratios of approximately 1:1 and 1:1, respectively [16]. These substitution values represent the average composition established for the parent DPSSC construct rather than a batch-specific molecular-weight measurement performed in the present study.

For the synthesis, DAB dendrimer (60 mg; 1 equivalent) was dissolved in DMSO (1.5 mL) and mixed for 10 min at 20 °C. OPSS-PEG-SCM (81.59 mg; 1 equivalent), freshly dissolved in a further 1.5 mL DMSO, was introduced dropwise over 10–12 min, after which the light-protected reaction proceeded for 6 h at 37 °C. Thiolated camptothecin (31 mg; 2 equivalents in 1.5 mL DMSO) was then incorporated, and stirring continued for 16 h at 37 °C in darkness. Purification was carried out at 20 °C in 3500 Da MWCO tubing, sequentially against DMSO (500 mL for 2 days), methanol (500 mL for 2 days), and distilled water (2 L for 2 days), replacing each medium twice per day. The recovered solution was passed through Whatman^®^ cellulose paper (18.5 cm diameter; 11 μm pores), lyophilized with a Christ Epsilon 2-4 LSC^®^ instrument (Osterode am Harz, Germany), and stored at 4 °C [16,31].

#### 2.4.3. Conjugation of Tf to DPSSC

**Figure 1:**
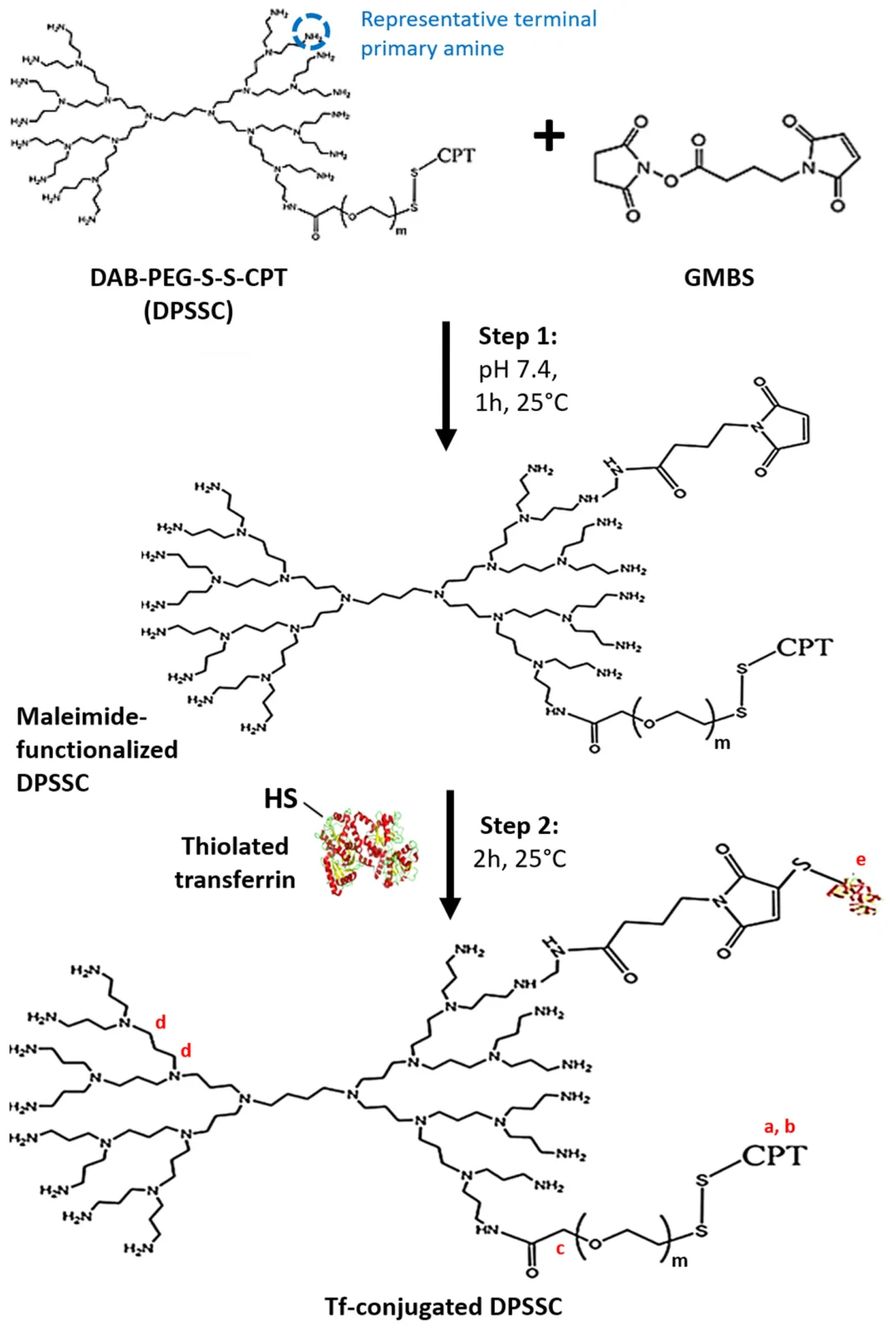
Stepwise covalent conjugation of Tf to DPSSC using GMBS. In step 1, the N-hydroxysuccinimide ester group of GMBS reacts with a representative terminal primary amino group of DPSSC to form an amide bond, leaving a thiol-reactive maleimide group on the dendrimer. In parallel, accessible primary amino groups of transferrin are modified with 2-iminothiolane hydrochloride to introduce free sulfhydryl groups, generating thiolated transferrin (Tf–SH). In step 2, Tf–SH reacts with maleimide-functionalized DPSSC through thiol–maleimide addition to form a covalent succinimidyl thioether linkage. The reaction scheme is representative and does not indicate a uniquely defined DPSSC or transferrin conjugation site. m denotes the average number of ethylene oxide repeat units in the PEG chain. a–e correspond to the subsequent ^1^H-NMR peak assignments.

DPSSC was conjugated to transferrin (Tf) using GMBS, a heterobifunctional cross-linker containing an amine-reactive N-hydroxysuccinimide ester and a thiol-reactive maleimide group, in a two-step procedure adapted from Hermanson [33]. In the first step, the N-hydroxysuccinimide ester of GMBS reacted with accessible terminal primary amino groups of DPSSC to form stable amide bonds, while leaving the maleimide functionality available for subsequent thiol coupling. The structure shown in Figure 1 represents one possible terminal amino-group modification for clarity. The reaction was not site-specific, and DPSSC retained multiple potentially reactive terminal amino groups.

In parallel, transferrin was reacted with 2-iminothiolane hydrochloride, also known as Traut’s reagent, to introduce free sulfhydryl groups through modification of accessible primary amino groups. The resulting thiolated transferrin, Tf–SH, was immediately reacted with maleimide-functionalized DPSSC. Thiol addition across the maleimide double bond produced a covalent succinimidyl thioether linkage between transferrin and DPSSC. The exact transferrin residue or modified residues and the orientation of transferrin on the conjugate were not determined.

DPSSC (20 mg) was solubilized in 2 mL of pH 7.4 buffer containing 50 mM sodium phosphate and 0.15 M sodium chloride. GMBS (6.6 mg in DMSO; a two-fold molar excess relative to DAB) was introduced, and the reaction was mixed for 1 h at 25 °C. The unreacted cross-linker was then removed by dialysis for 24 h at 20 °Cin benzoylated tubing (MWCO 2000 Da) against 500 mL of the same 50 mM sodium phosphate/0.15 M sodium chloride buffer, which was replaced twice [31].

For thiolation, 20 mg Tf was dissolved in 2 mL of sodium phosphate/sodium chloride buffer (50 mM/0.15 M, pH 8.0). A ten-fold molar excess of Traut’s reagent was added (153 μL of a 2 mg/mL aqueous solution; 14.5 mM), and the mixture was held for 1 h at 20 °C. Tf-SH was isolated in a Vivaspin-4^®^ concentrator (MWCO 5000 Da; 15 min at 6288× *g*) and immediately mixed with maleimide-functionalized DPSSC for 2 h at 25 °C. A second Vivaspin-4^®^ separation under the same conditions removed unconjugated dendrimer. The conjugate was subsequently dialyzed for 24 h at 20 °C in SnakeSkin^®^ tubing (MWCO 3500 Da) against 1 L distilled water, with one medium change, before freeze-drying on a Christ Epsilon 2-4 LSC^®^ system (Osterode am Harz, Germany) [31].

Based on the previously determined average molecular weight of approximately 4480 Da for DPSSC and an approximate molecular weight of 79.5 kDa for human transferrin, the 20 mg quantities used corresponded to approximately 4.46 µmol DPSSC and 0.25 µmol transferrin. The nominal Tf:DPSSC reaction-feed ratio was therefore approximately 0.056:1 mol/mol. This value represents the initial reaction-feed ratio and not the experimentally measured post-purification conjugation ratio.

### 2.5. Structural Elucidation

Transferrin (Tf)-conjugated DPSSC (3 mg dissolved in 0.5 mL CDCl_3_) was characterized by proton nuclear magnetic resonance (^1^H NMR) spectroscopy using a Bruker Avance^®^ III-HD-500 spectrometer (Billerica, MA, USA). The residual proton signal of the deuterated solvent was used as the internal chemical shift reference.

### 2.6. Evaluation of Self-Assembling Ability

The self-assembly of the amphiphilic transferrin (Tf)-conjugated DPSSC dendrimer in phosphate-buffered saline (PBS, pH 7.4) was evaluated by steady-state fluorescence spectroscopy using Nile red as a hydrophobic probe. Nile red exhibits weak fluorescence in aqueous media but shows markedly increased fluorescence intensity upon partitioning into non-polar environments, such as the hydrophobic core of self-assembled nanostructures.

Nile red stock (16 μL; 3.14 × 10^−3^ M in methanol, prepared from 0.98 mg in 0.98 mL) was placed in 5 mL plastic vials. Tf-conjugated DPSSC solutions covering 0.05–3500 μg/mL in PBS (pH 7.4) were added, vortexed for 5 min, and protected from light. Spectra were acquired with a Varian Cary Eclipse^®^ fluorometer using an excitation at 600 nm, an emission range of 620–800 nm, and excitation and emission slit widths of 5 nm. The critical aggregation concentration was assigned from the change in slope of relative Nile red fluorescence at λ_max_ versus dendrimer concentration [31].

### 2.7. Morphology

Vesicle morphology was visualized by TEM on a JEOL JEM-1200EX^®^ microscope (JEOL, Tokyo, Japan) operated at 80 kV. A 3 μL portion of Tf-conjugated DPSSC suspension was applied to a 400-mesh carbon-coated copper grid, excess liquid was blotted away, and the grid was left overnight in a desiccator before imaging [31].

### 2.8. Entrapment of Docetaxel

Docetaxel (32 μg in methanol) was encapsulated in Tf-bearing DPSSC dendrimersomes (4 mg dendrimer in 1993.6 μL PBS) by probe sonication using a Soniprep^®^ 150 sonicator (MSE, Heathfield, UK). Sonication was performed for five cycles of 2 min each. The resulting suspension was centrifuged at 14,000× *g* for 20 min at 20 °C using an Avanti^®^ J-E centrifuge (Beckman Coulter, London, UK). Unencapsulated docetaxel was removed by washing the pellet with 2 mL ultrapure water followed by a second centrifugation under identical conditions. The final dendrimersome suspension was adjusted to 1 mL with ultrapure water and stored at 4 °C until use.

Docetaxel entrapment and covalently associated camptothecin were measured at 312 and 432 nm, respectively, with an Agilent Varian Cary^®^ 50 spectrophotometer (Agilent Technologies, Santa Clara, CA, USA). Before analysis, a 10 μL formulation was diluted with 990 μL isopropanol and subjected to five 2 min sonication cycles to disassemble the vesicles [31].

### 2.9. pH-Dependent Drug Release

The pH-dependent release of docetaxel and camptothecin from Tf-bearing DPSSC dendrimersomes was evaluated at pH 7.4, 6.5 and 5.5. These values were selected to represent physiological blood and normal extracellular conditions (pH 7.4), the mildly acidic extracellular tumor microenvironment and early endosomal conditions (pH 6.5), and the more acidic late endosomal/upper lysosomal environment encountered following cellular internalization (pH 5.5). The pH 5.5 condition was not intended to represent the cytosol, which remains approximately neutral.

Tf-bearing DPSSC dendrimersomes containing 500 μg docetaxel were sealed in 3.5 kDa MWCO SnakeSkin^®^ dialysis tubing (Thermo Fisher Scientific, Waltham, MA, USA) and immersed in 50 mL PBS adjusted to the required pH. Release was conducted at 37 °C with continuous stirring [31].

Triplicate 1 mL samples were withdrawn from the receiving phase at 0.5 h, hourly from 1 to 6 h, and at 8, 10, 12, and 24 h. Each withdrawn volume was replaced by a fresh buffer of identical pH. Absorbance at 312 nm for docetaxel and 432 nm for camptothecin was used to calculate transfer into the dialysate, and cumulative release was expressed as a percentage of total drug content [31].

### 2.10. Redox-Dependent Drug Release

Redox-triggered release of docetaxel and camptothecin from Tf-conjugated DPSSC dendrimersomes was evaluated in the absence of glutathione (GSH) and in the presence of 10 µM, 10 mM or 50 mM GSH. The 10 µM condition was selected to approximate the relatively low extracellular and plasma GSH environment, whereas 10 mM represented the millimolar reducing environment of the intracellular cytosol, including that of many cancer cells. The 50 mM concentration was included as a strongly reducing accelerated challenge condition to assess the maximal susceptibility of the disulfide-containing formulation to reductive cleavage and was not intended to reproduce average physiological GSH concentrations.

Tf-conjugated DPSSC dendrimersomes entrapping docetaxel were diluted in PBS (pH 7.4) to a final dendrimer concentration of 1 mg/mL and incubated with GSH at final concentrations of 0, 10 μM, 10 mM or 50 mM. Samples (final volume 1 mL) were prepared as follows:

- 0 mM GSH: 200 μL dendrimersomes (5 mg/mL) + 800 μL PBS;
- 10 μM GSH: 200 μL dendrimersomes (5 mg/mL) + 700 μL PBS + 100 μL GSH (100 μM);
- 10 mM GSH: 200 μL dendrimersomes (5 mg/mL) + 700 μL PBS + 100 μL GSH (100 mM);
- 50 mM GSH: 200 μL dendrimersomes (5 mg/mL) + 300 μL PBS + 500 μL GSH (100 mM).

The 1 mL preparations were enclosed in 3.5 kDa MWCO tubing and placed in 60 mL PBS (pH 7.4) containing the same GSH concentration, thereby maintaining the chosen reducing environment on both sides of the membrane. Dialysis proceeded in darkness at 37 °C with continuous mixing for up to 7 days [31].

At each sampling point, three 1 mL portions were removed from the external phase and replaced with fresh, pre-equilibrated medium. Calibration curves in pH 7.4 PBS and absorbance readings at 312 nm for docetaxel and 432 nm for camptothecin were used for quantification. Cumulative release was normalized to the total amount of each drug [31].

### 2.11. Cellular Uptake

#### 2.11.1. Qualitative Analysis

Cellular uptake of Tf-conjugated DPSSC dendrimersomes encapsulating fluorescein-labeled docetaxel was qualitatively assessed by confocal laser scanning microscopy. PC3-Luc, DU145, and LNCaP cells were plated onto coverslips in 6-well dishes at 2 × 10^5^ cells per well in 3 mL complete medium and incubated for 24 h at 37 °C. Fresh medium containing Tf-conjugated DPSSC, non-targeted DPSSC, free docetaxel, or free camptothecin was then applied, with drug exposure matched at 0.9 μg docetaxel and 7.38 μg camptothecin per well (1:8 molar ratio). Four hours later, cells were rinsed three times with pH 7.4 PBS, fixed for 10 min at 20 °C with 2 mL methanol, and air-dried for 5 min. Coverslips were mounted in propidium iodide-containing Vectashield^®^ and imaged 2 h later on a Leica TCS SP5^®^ confocal microscope (Leica Microsystems, Wetzlar, Germany). Propidium iodide was excited at 543 nm (emission 597–647 nm), camptothecin at 405 nm (emission 400–480 nm), and fluorescein-labeled docetaxel at 494 nm (emission 510–530 nm) [31].

#### 2.11.2. Quantitative Analysis

Cellular uptake of fluorescently labeled docetaxel was also quantified by flow cytometry. PC3-Luc, DU145, and LNCaP cells were plated in 6-well plates and exposed under the same conditions used for confocal imaging. After 4 h, the treatment medium was discarded, each well was washed three times with 2 mL pH 7.4 PBS, and cells were released with 250 μL trypsin before resuspension in a 500 μL complete medium to obtain single-cell suspensions.

Samples were acquired on an Attune^®^ NxT Acoustic Focusing flow cytometer and processed with Attune^®^ NxT software (Thermo Fisher Scientific, Waltham, MA, USA). For every sample, 10,000 gated events were collected, and cellular fluorescence intensity was used as the uptake readout [31].

#### 2.11.3. Mechanisms of Cellular Uptake

The mechanisms involved in the cellular uptake of fluorescein-labeled docetaxel encapsulated in Tf-conjugated DPSSC dendrimersomes were investigated using pharmacological inhibitors of endocytic pathways. PC3-Luc cells were seeded and cultured as described above. After removal of the culture medium, cells were exposed for 15 min at 37 °C to DMSO-prepared free transferrin (80 μM), phenylarsine oxide (10 μM), filipin (5 μg/mL), colchicine (10 μM), or poly-L-lysine (40 μg/mL). Without removing the inhibitor, docetaxel-loaded Tf-conjugated DPSSC was added to provide 0.9 μg docetaxel and 7.38 μg camptothecin per well (1:8 molar ratio), and incubation continued for 4 h at 37 °C. Cells were then washed three times with 2 mL PBS and evaluated by the confocal and flow-cytometric procedures described above [31].

### 2.12. Anti-Proliferative Efficacy

The anti-proliferative activity of Tf-conjugated DPSSC dendrimersomes encapsulating docetaxel was assessed using an MTT assay. PC3-Luc, DU145, and LNCaP cells were seeded into 96-well plates at 5000 cells/well and cultured for 48 h at 37 °C. They were then exposed to docetaxel-loaded Tf-conjugated DPSSC, the non-targeted DPSSC control, docetaxel and camptothecin solutions either separately or together, with all relevant conditions standardized to 0.9 μg docetaxel and 7.38 μg camptothecin per well (1:8 molar ratio). Untreated wells and 50 μL/well Triton X-100 (1% *w*/*v*) were used as negative and positive controls. Following 48 h of treatment, 50 μL of 0.5% (*w*/*v*) MTT in PBS was added for 4 h at 37 °C in darkness. After aspiration, the formazan product was dissolved in 200 μL DMSO per well and absorbance was read at 570 nm with a Multiskan^®^ Ascent plate reader (Thermo Labsystems, Beverly, MA, USA). Viability was normalized to untreated cells. IC_50_ values were obtained in OriginPro^®^ 2021 (OriginLab, Northampton, MA, USA) by fitting viability against log concentration [31].

### 2.13. Statistical Analysis

All data are presented as mean ± standard error of the mean (SEM). Statistical analyses were performed by one-way analysis of variance (ANOVA) followed by Tukey’s post hoc test for multiple comparisons using Minitab^®^ 19 (State College, PA, USA). Differences were considered statistically significant at *p* < 0.05.

## 3. Results

### 3.1. Drug Synergy

**Figure 2:**
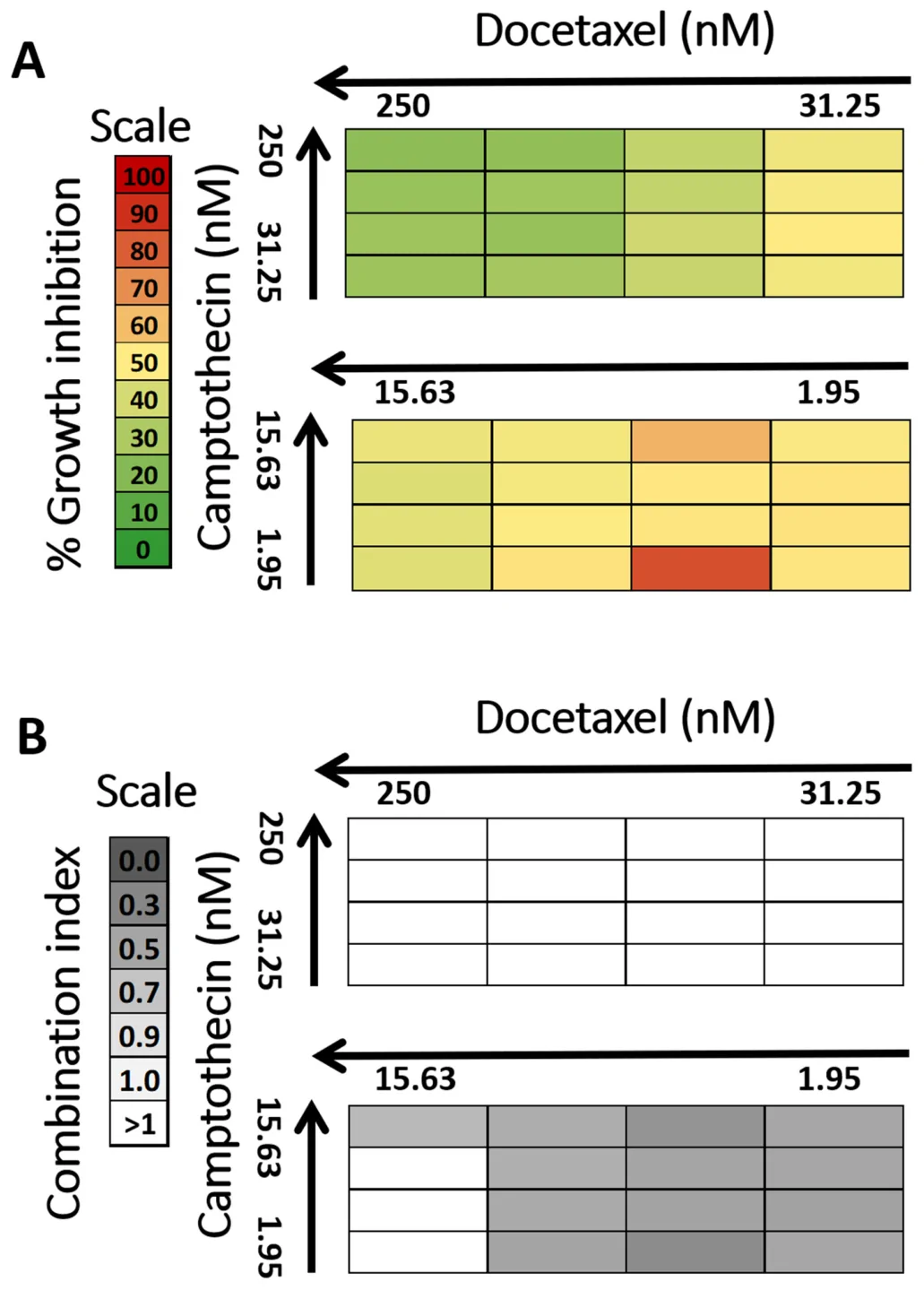
Evaluation of the synergistic interaction between docetaxel and camptothecin over a concentration range of 250 to 1.95 nM. (**A**) Percentage growth inhibition (%GI) of PC3-Luc cells treated with different combinations of docetaxel and camptothecin, determined by MTT assay (n = 15). Red indicates %GI > 80%, orange indicates %GI of 60–80%, yellow indicates %GI of 50–60%, and green indicates %GI < 50%. (**B**) Combination index (CI) values calculated using CompuSyn^®^ software for the corresponding drug combinations (*n* = 15). Dark grey indicates a highly synergistic effect (CI < 0.3); grey indicates a synergistic effect (0.3 < CI < 1); light grey indicates an additive effect (CI = 1); and white indicates an antagonistic effect (CI > 1).

The synergistic interaction between docetaxel and camptothecin in PC3-Luc cells was strongly dependent on the concentration combination used (Figure 2). Overall, the highest concentrations tested were associated with relatively modest growth inhibition and predominantly antagonistic effects, with combination index (CI) values ranging from 1.39 ± 0.13 to 2.50 ± 0.12 and growth inhibition values between 22.48 ± 0.74% and 49.71 ± 0.36%. In contrast, the lower concentration combinations produced markedly improved responses, with several combinations showing clear synergistic effects and higher growth inhibition values. The most pronounced synergy was observed for the combination of 3.91 nM docetaxel and 1.95 nM camptothecin, which gave a CI of 0.20 ± 0.01 together with the highest growth inhibition of 88.10 ± 0.41%. The second strongest synergistic interaction was obtained at 3.91 nM docetaxel and 15.63 nM camptothecin, with a CI of 0.32 ± 0.02 and a growth inhibition of 72.62 ± 1.56%. Other low-concentration combinations also showed synergistic effects, with CI values between 0.54 ± 0.14 and 0.75 ± 0.08, generally accompanied by growth inhibition values of approximately 61–66%. These results indicate that the docetaxel/camptothecin combination was most effective in the lower nanomolar range, with the strongest anti-proliferative effect and the greatest degree of synergy achieved at 3.91 nM docetaxel combined with 1.95 nM or 15.63 nM camptothecin. Because the camptothecin concentration corresponding to the strongest synergistic condition was difficult to use in the synthesis of the dendrimersomes, the combination of 3.91 nM docetaxel and 15.63 nM camptothecin was selected for the follow-up experiments.

### 3.2. Synthesis of Transferrin-Conjugated, Disulfide-Linked Camptothecin-Bearing PEGylated DAB Dendrimer (Tf-Conjugated DPSSC)

**Figure 3:**
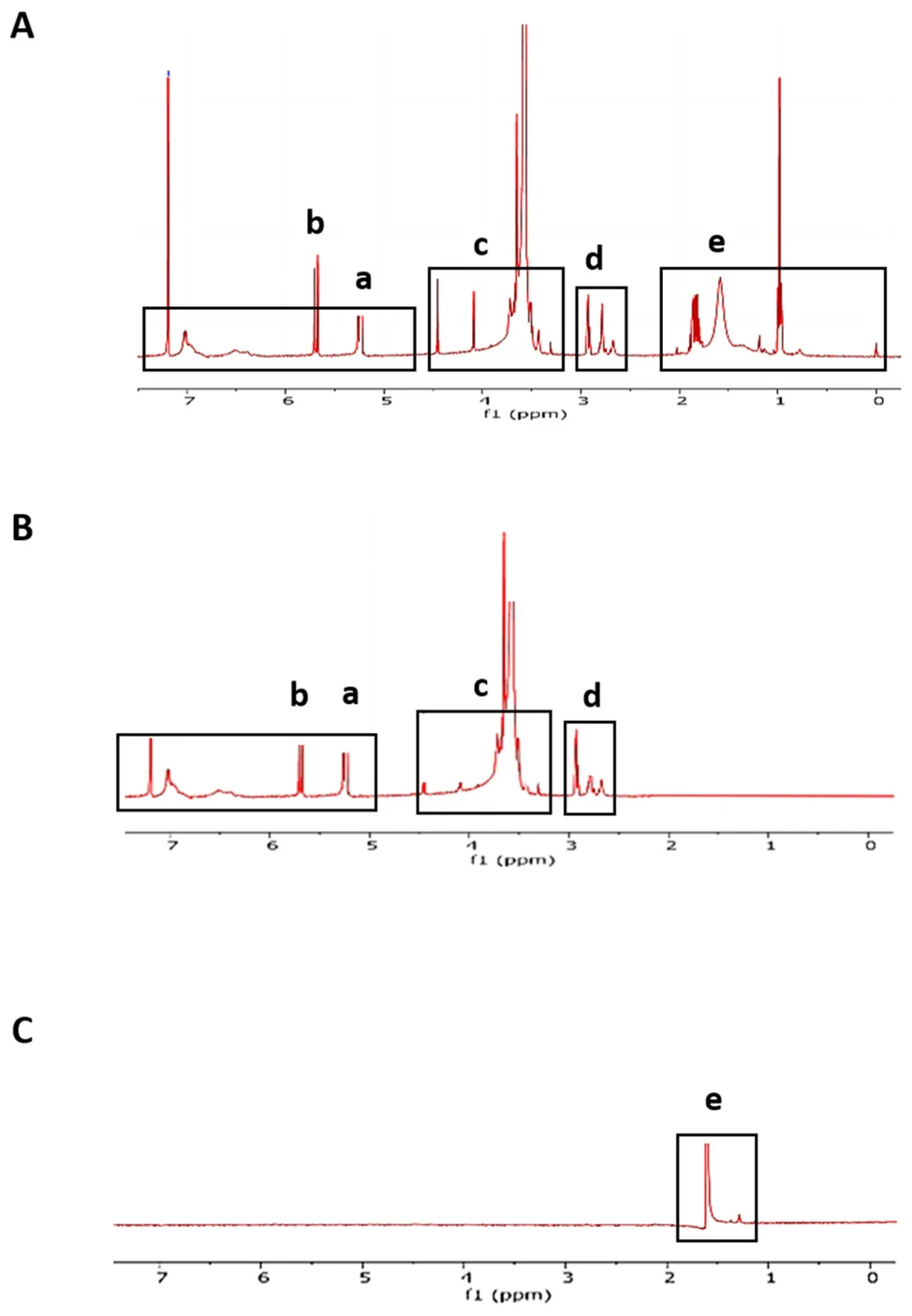
^1^H-NMR spectra (500 MHz, CDCl_3_) of Tf-conjugated camptothecin-bearing PEGylated DAB dendrimer (Tf–DPSSC) (**A**), camptothecin-bearing PEGylated DAB dendrimer (DPSSC) (**B**), and Tf (**C**). Peak assignments: (a,b) camptothecin, (c) PEG, (d) DAB, and (e) Tf.

Successful synthesis of Tf-bearing DPSSC was confirmed by ^1^H-NMR spectroscopy (500 MHz, CDCl_3_). The spectrum of Tf-conjugated DPSSC displayed the characteristic resonances of camptothecin at δ 5.24–5.27 ppm (CH–**CH**–N–CO, a) and δ 5.65–5.71 ppm (CH–**CH**–O, b), alongside signals assigned to the PEG chain at δ 3.82–3.90 ppm (NH–CO–**CH_2_**–OCH_2_CH_2_, c) and the DAB dendrimer backbone at δ 2.48–2.59 ppm (N**CH_2_–**CH_2_–**CH_2_**–N, d). Importantly, a distinct resonance at δ 1.50 ppm (e), matching the Tf reference spectrum, was observed in Tf-conjugated DPSSC but was absent from DPSSC alone, confirming successful Tf conjugation (Figure 3) [31].

### 3.3. Evaluation of Self-Assembling Ability

**Figure 4:**
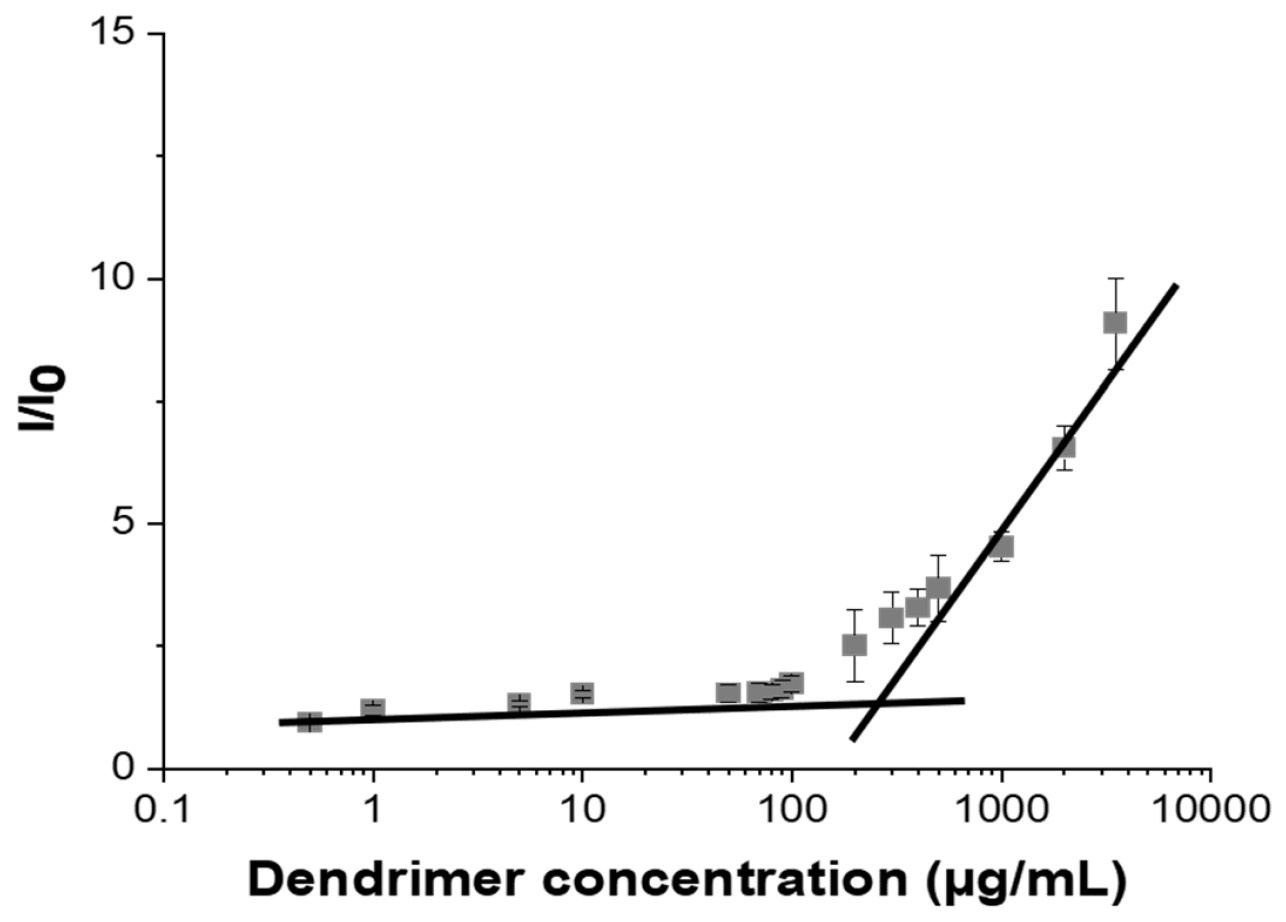
Relative fluorescence intensity (I/I_0_) of Nile red as a function of Tf–DPSSC concentration in PBS (pH 7.4) (*n* = 3). The CAC was determined from the inflection point of the fluorescence–concentration plot.

The self-assembly of Tf-bearing DPSSC in PBS (pH 7.4) was evaluated using Nile red fluorescence. As a highly hydrophobic probe, Nile red is only weakly fluorescent in aqueous media but becomes strongly fluorescent when partitioned into non-polar environments, such as the hydrophobic domains formed upon dendrimer aggregation. Accordingly, the relative fluorescence intensity (I/I_0_) remained close to the baseline at low Tf–DPSSC concentrations, and then increased sharply above a threshold concentration, consistent with the formation of self-assembled aggregates providing a hydrophobic microenvironment for Nile red (Figure 4). The critical aggregation concentration (CAC), determined from the inflection point (intersection of the low- and high-concentration linear regions), was estimated to be approximately 250 µg/mL [31].

### 3.4. Morphology

**Figure 5:**
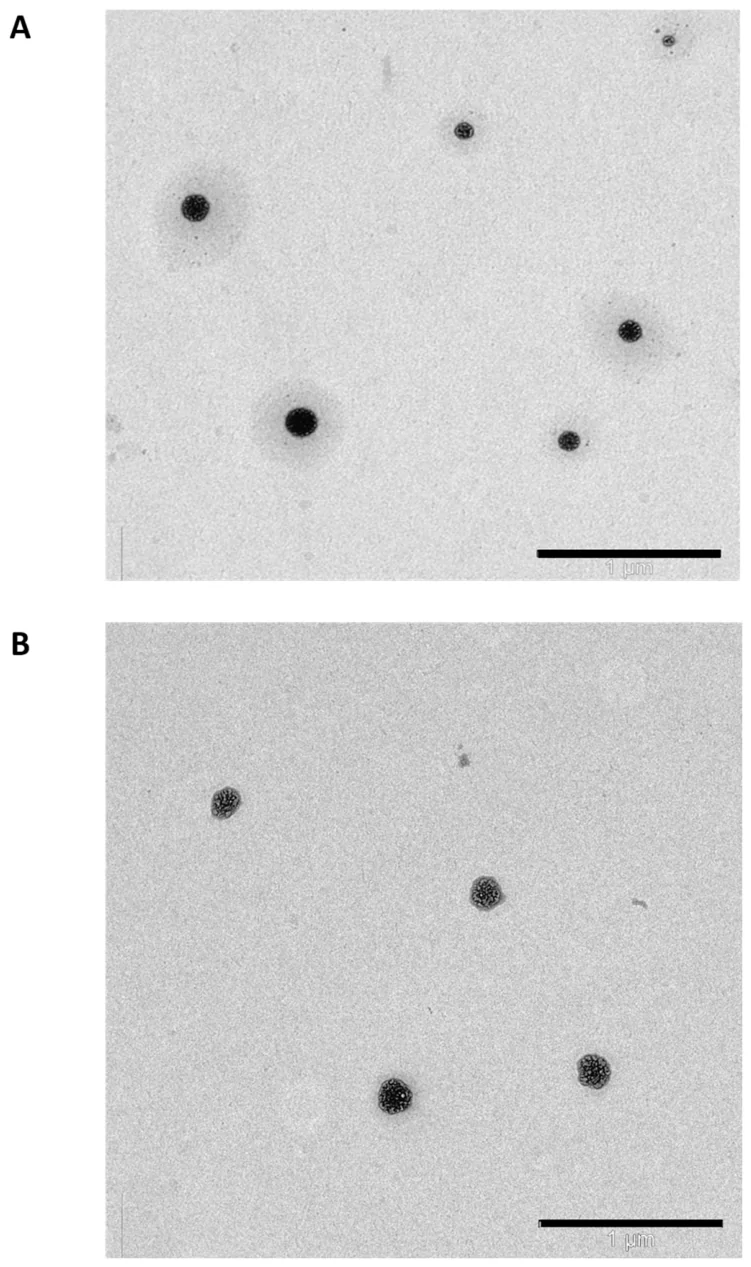
TEM micrographs of Tf-conjugated DPSSC dendrimersomes (**A**) and DPSSC dendrimersomes (**B**) in PBS (pH 7.4) at a concentration of 500 µg/mL (scale bar: 1 µm).

TEM analysis confirmed that Tf-conjugated DPSSC self-assembled in PBS (pH 7.4) into spherical vesicular nanostructures (dendrimersomes) (Figure 5). The particles appeared well dispersed with minimal aggregation and remained below one micrometer in the dry-state images [31].

### 3.5. Entrapment of Docetaxel

The DTX entrapment efficiency of Tf-conjugated DPSSC dendrimersomes was 89.10 ± 0.08%. Based on an initial DTX input of 32 µg, this corresponded to approximately 28.51 µg DTX recovered in the complete 1 mL purified formulation, equivalent to approximately 28.51 µg/mL or 0.285 µg in the 10 µL aliquots used for analysis. The calculated unrecovered amount was approximately 3.49 µg.

The CPT amount measured in the 10 µL analytical aliquot was 2.37 ± 0.04 µg, corresponding to approximately 237 µg/mL and 237 µg in the complete 1 mL formulation. The corresponding conjugation efficiency of camptothecin to the DAB dendrimer was 10.90 ± 0.05%.

### 3.6. pH-Dependent Drug Release

**Figure 6:**
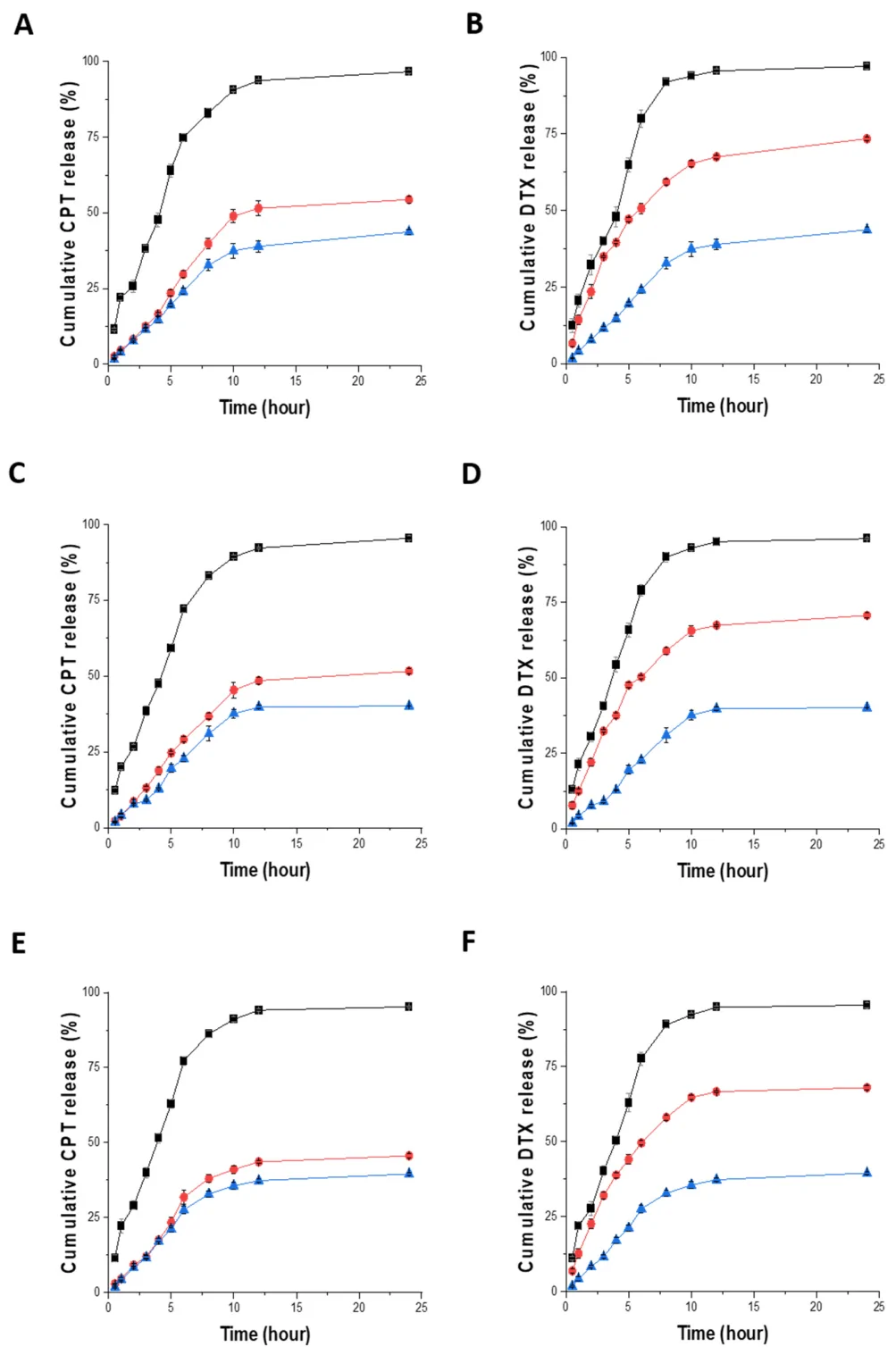
In vitro release profiles of docetaxel (**A**,**C**,**E**) and camptothecin (**B**,**D**,**F**) formulated as Tf-conjugated DPSSC (●, red), control DPSSC (▲, blue), or free drug solution (■, black), determined by dialysis against 50 mL PBS over 24 h at 37 °C. Release profiles were evaluated at pH 7.4 (**A**,**B**), pH 6.5 (**C**,**D**), and pH 5.5 (**E**,**F**) (*n* = 3). Error bars were smaller than the symbols when not visible.

The release profiles of docetaxel and camptothecin from Tf-conjugated DPSSC were evaluated at pH 7.4, 6.5, and 5.5 using a dialysis method. For docetaxel, an initial burst release corresponding to approximately half of the total released drug was observed within the first 5 h, followed by a more sustained release over the subsequent 10 h, reaching maximum cumulative release by 24 h. The highest cumulative release of docetaxel from Tf-conjugated camptothecin-based dendrimersomes was observed at pH 5.5 (73.46 ± 0.61%), followed by pH 6.5 (70.69 ± 0.83%) and pH 7.4 (65.06 ± 0.75%). In contrast, free docetaxel solution showed significantly higher cumulative release percentages at all pH values, reaching 95.72 ± 0.49% at pH 5.5, 94.84 ± 0.43% at pH 6.5, and 94.45 ± 0.38% at pH 7.4 (Figure 6). Camptothecin showed a similar trend, although its cumulative release percentages were slightly lower than those of docetaxel. The maximum cumulative release of camptothecin from Tf-conjugated DPSSC was recorded at pH 5.5 (50.67 ± 1.20%), followed by pH 6.5 (49.33 ± 1.20%) and pH 7.4 (42.87 ± 1.74%). As observed for docetaxel, free camptothecin solution exhibited significantly higher cumulative release percentages, reaching 94.81 ± 0.47% at pH 5.5, 94.24 ± 0.38% at pH 6.5, and 93.71 ± 0.18% at pH 7.4 (Figure 6) [31].

### 3.7. Redox-Dependent Drug Release

**Figure 7:**
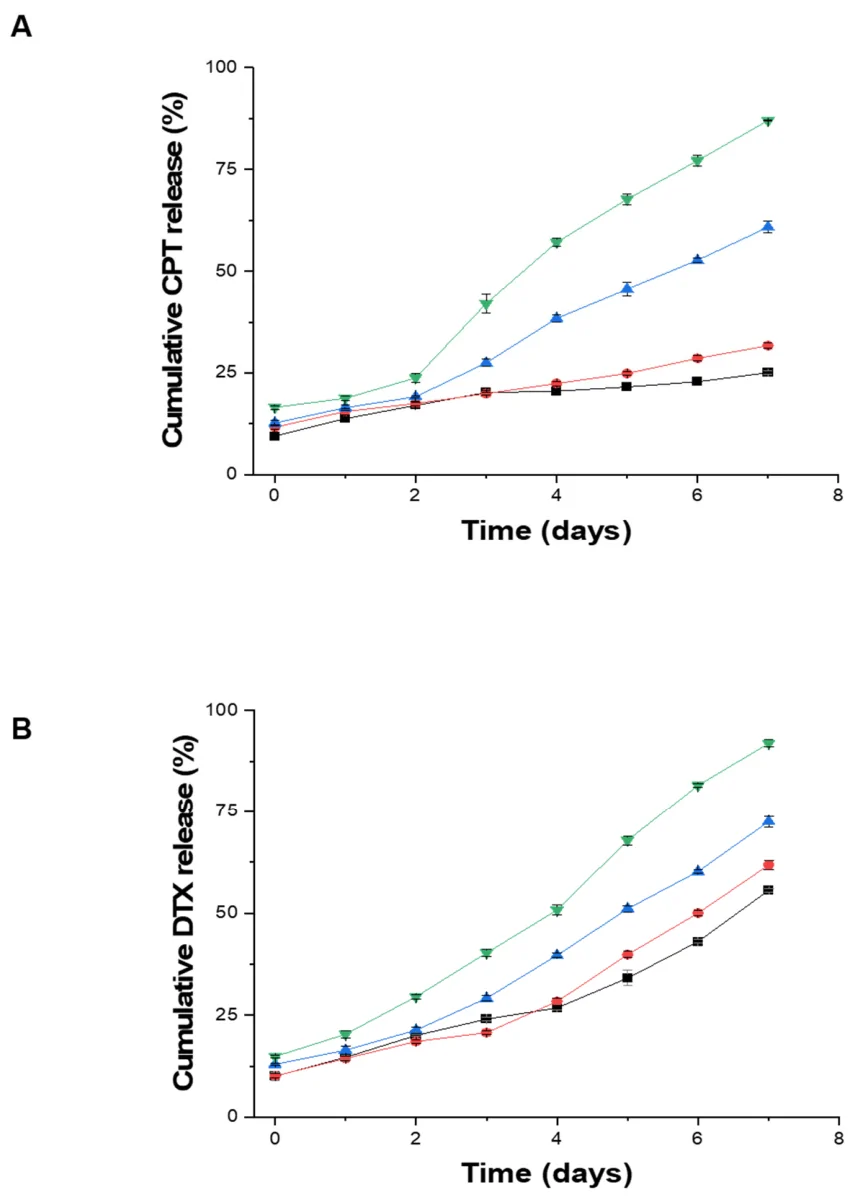
Cumulative release profiles of camptothecin (**A**) and docetaxel (**B**) from Tf-conjugated DPSSC (1 mg/mL in PBS, pH 7.4), measured over 7 days at 37 °C. Data were obtained in the presence of 50 mM GSH (▼, green), 10 mM GSH (▲, blue), 10 µM GSH (●, red), and in the absence of GSH (■, black) (*n* = 3). Error bars are smaller than the symbols when not visible.

In the absence of GSH, Tf-conjugated DPSSC vesicles showed only minimal camptothecin release, with a cumulative release of 24.52 ± 0.58% after 7 days of incubation. Release remained relatively low in the presence of 10 μM GSH (31.26 ± 0.63%), but increased markedly at higher GSH concentrations, reaching 60.48 ± 1.25% at 10 mM GSH and 86.85 ± 0.57% at 50 mM GSH (Figure 7A). A similar trend was observed for docetaxel, whose cumulative release after 7 days increased from 55.33 ± 0.88% in the absence of GSH to 61.66 ± 0.88%, 72.30 ± 0.74%, and 91.63 ± 0.46% in the presence of 10 μM, 10 mM, and 50 mM GSH, respectively (Figure 7B).

### 3.8. Cellular Uptake

#### 3.8.1. Qualitative Analysis

**Figure 8:**
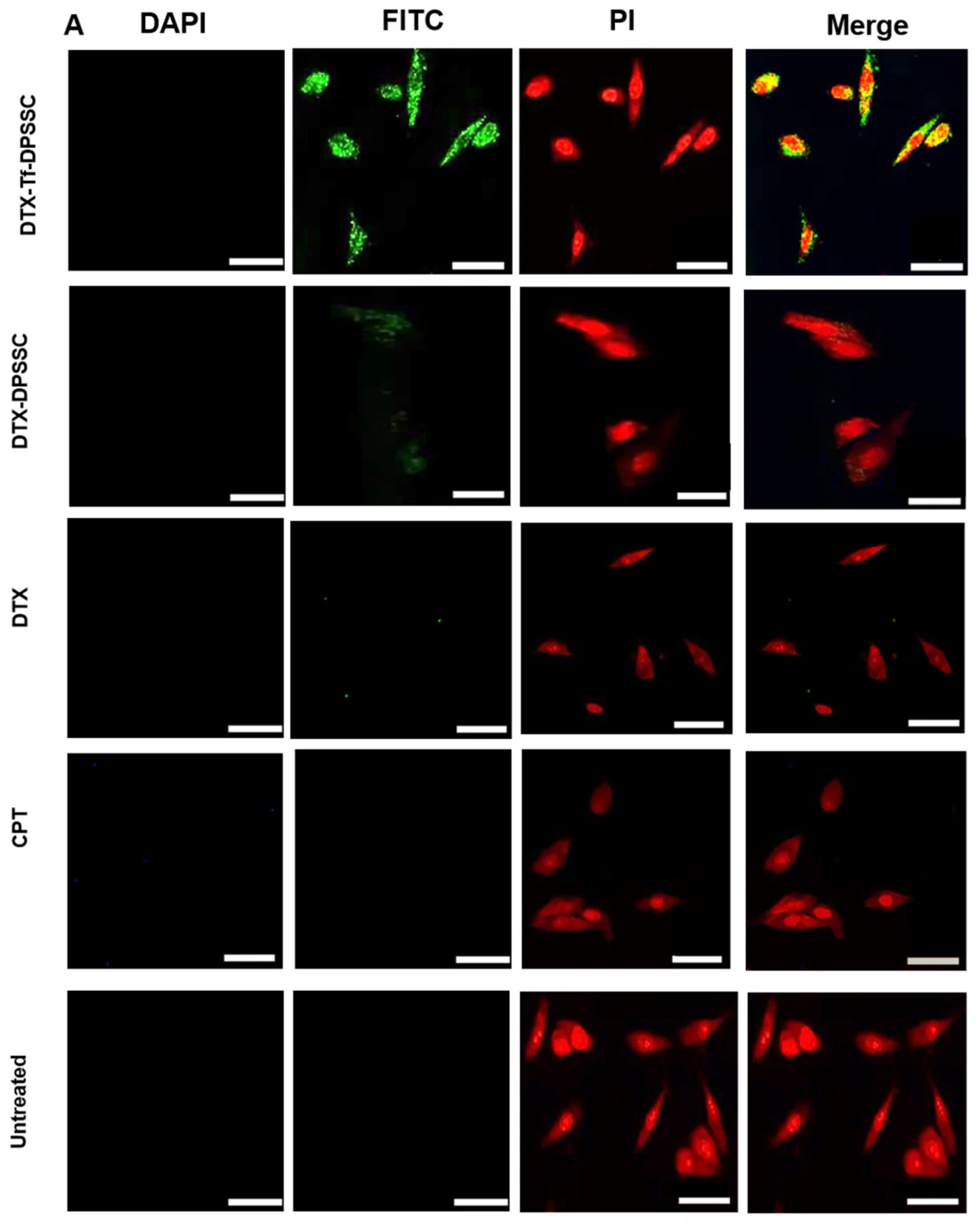

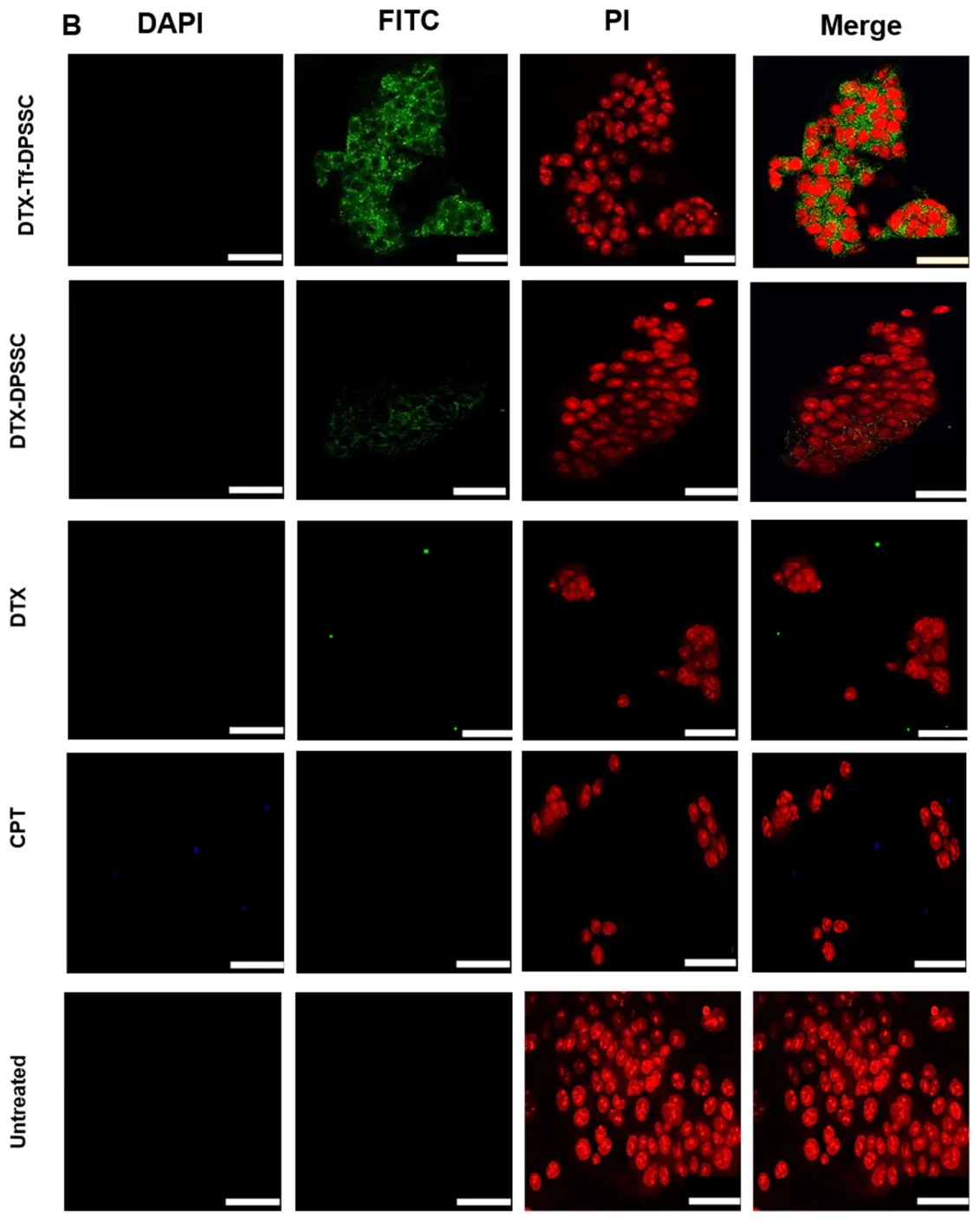

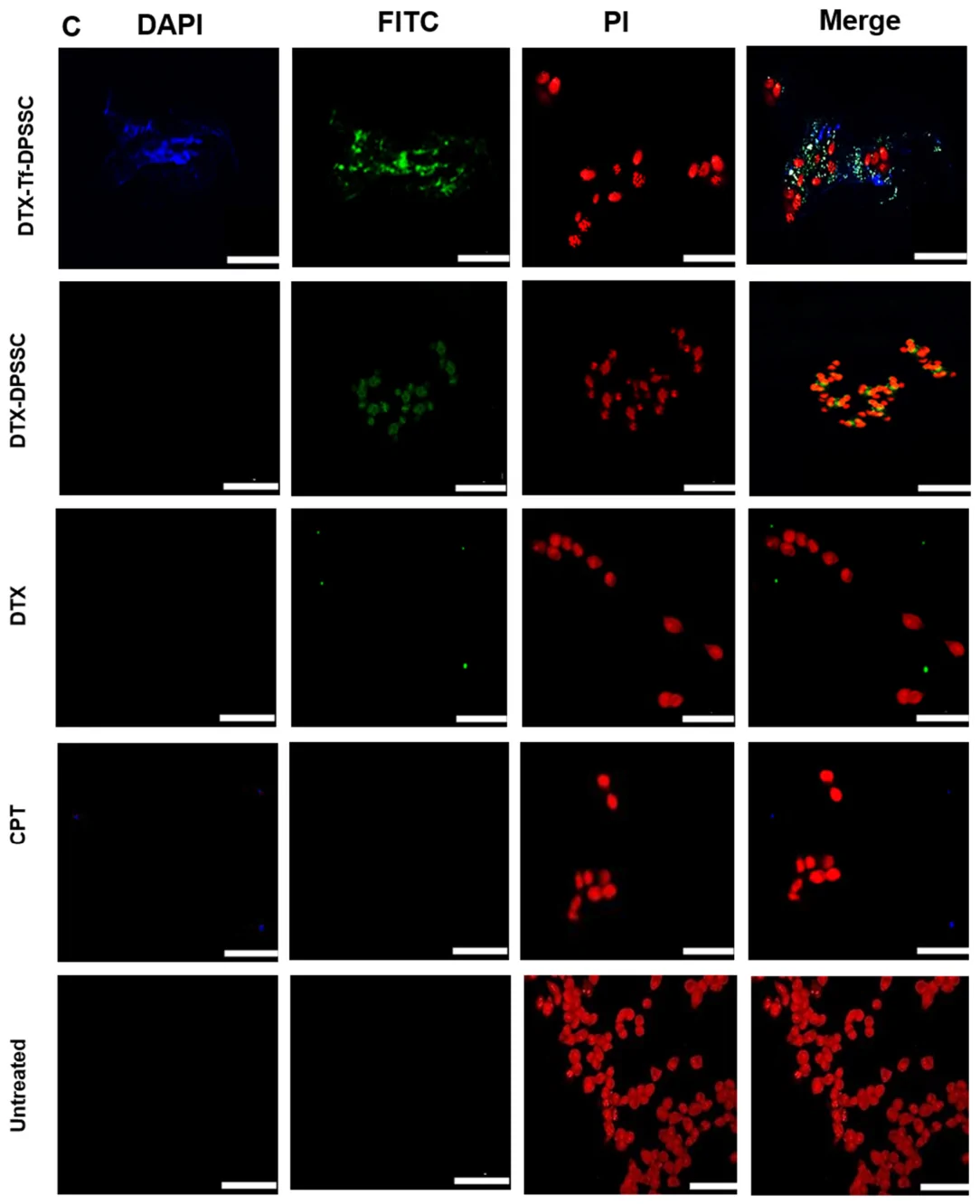
Confocal microscopy images of the cellular uptake of fluorescein-labeled docetaxel delivered as Tf-conjugated DPSSC (DTX-Tf-DPSSC), non-targeted DPSSC (DTX-DPSSC), or free drug solutions in PC3-Luc (**A**), DU145 (**B**), and LNCaP (**C**) prostate cancer cells following 4 h incubation at 37 °C. Red fluorescence corresponds to nuclei stained with propidium iodide (excitation: 543 nm; emission: 597–647 nm), green fluorescence to fluorescein-labeled docetaxel (excitation: 494 nm; emission: 517 nm), and blue fluorescence to camptothecin (excitation: 405 nm; emission: 400–480 nm). Scale bar = 50 µm.

The cellular uptake of fluorescein-labeled docetaxel encapsulated in Tf-conjugated DPSSC dendrimersomes was qualitatively evaluated in PC3-Luc, DU145, and LNCaP prostate cancer cells using confocal microscopy. Intense fluorescence was observed in the cytoplasm of all three cell lines, indicating efficient intracellular uptake of docetaxel, and slight nuclear diffusion was additionally noted in PC3-Luc cells following treatment with Tf-conjugated DPSSC. By contrast, camptothecin fluorescence was observed only in the cytoplasm of LNCaP cells (Figure 8A–C). Cells treated with non-targeted DPSSC containing docetaxel displayed lower fluorescence intensity than those treated with the Tf-conjugated formulation, consistent with reduced uptake. Minimal fluorescence was detected in cells treated with free docetaxel or camptothecin solutions, further supporting the enhanced uptake of the dendrimersome formulations compared with the corresponding free drugs (Figure 8A–C) [31].

#### 3.8.2. Quantitative Analysis

**Figure 9:**
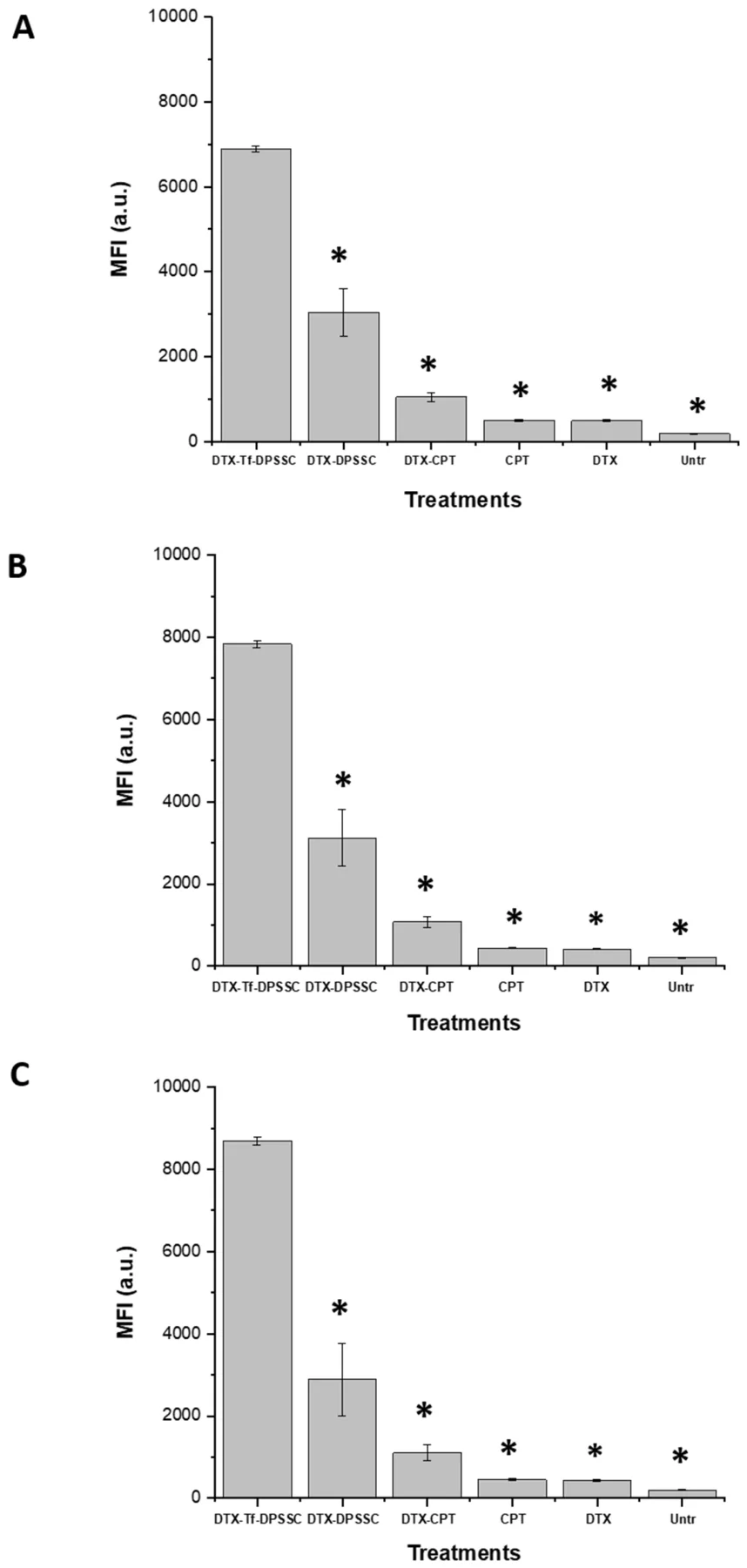
Flow cytometry analysis of the cellular uptake of fluorescein-labeled docetaxel delivered as Tf-conjugated DPSSC, control DPSSC, or free drug solutions in PC3-Luc (**A**), DU145 (**B**), and LNCaP (**C**) prostate cancer cells after 4 h of incubation (*n* = 5). *: *p* < 0.05 compared with Tf-conjugated DPSSC loading docetaxel.

The cellular uptake of fluorescein-labeled docetaxel entrapped in Tf-conjugated DPSSC was quantitatively evaluated by flow cytometry. In all three prostate cancer cell lines, namely PC3-Luc, DU145, and LNCaP, treatment with Tf-conjugated DPSSC loading docetaxel produced the highest cellular fluorescence compared with control DPSSC loading docetaxel, a combination of docetaxel and camptothecin solutions, and the individual docetaxel and camptothecin solutions. In PC3-Luc cells, the mean fluorescence intensity (MFI) of fluorescein-labeled docetaxel delivered by Tf-conjugated DPSSC reached 6890 ± 68 arbitrary units (a.u.), corresponding to a 2.27-fold increase relative to DPSSC loading docetaxel (3035 ± 566 a.u.) and a 17.3-fold increase relative to docetaxel solution (482 ± 20 a.u.) (Figure 9A). A similar trend was observed in DU145 cells, in which Tf-conjugated DPSSC loading docetaxel yielded the highest fluorescence intensity (7834 ± 90 a.u.), representing a 2.51-fold and 18.8-fold increase compared with DPSSC loading docetaxel (3121 ± 690 a.u.) and docetaxel solution (416 ± 15 a.u.), respectively (Figure 9B). The highest MFI overall was recorded in LNCaP cells, where fluorescein-labelled docetaxel delivered by Tf-conjugated DPSSC reached 8693 ± 100 a.u., corresponding to a 3.01-fold increase relative to DPSSC loading docetaxel (2883 ± 890 a.u.) and a 20-fold increase relative to docetaxel solution (434 ± 18 a.u.) (Figure 9C). These findings demonstrate a marked enhancement in cellular uptake mediated by the Tf-conjugated formulation, supporting the potential of Tf-conjugated DPSSC as an effective nanocarrier for the delivery of docetaxel and camptothecin to prostate cancer cells.

#### 3.8.3. Mechanisms of Cellular Uptake

**Figure 10:**
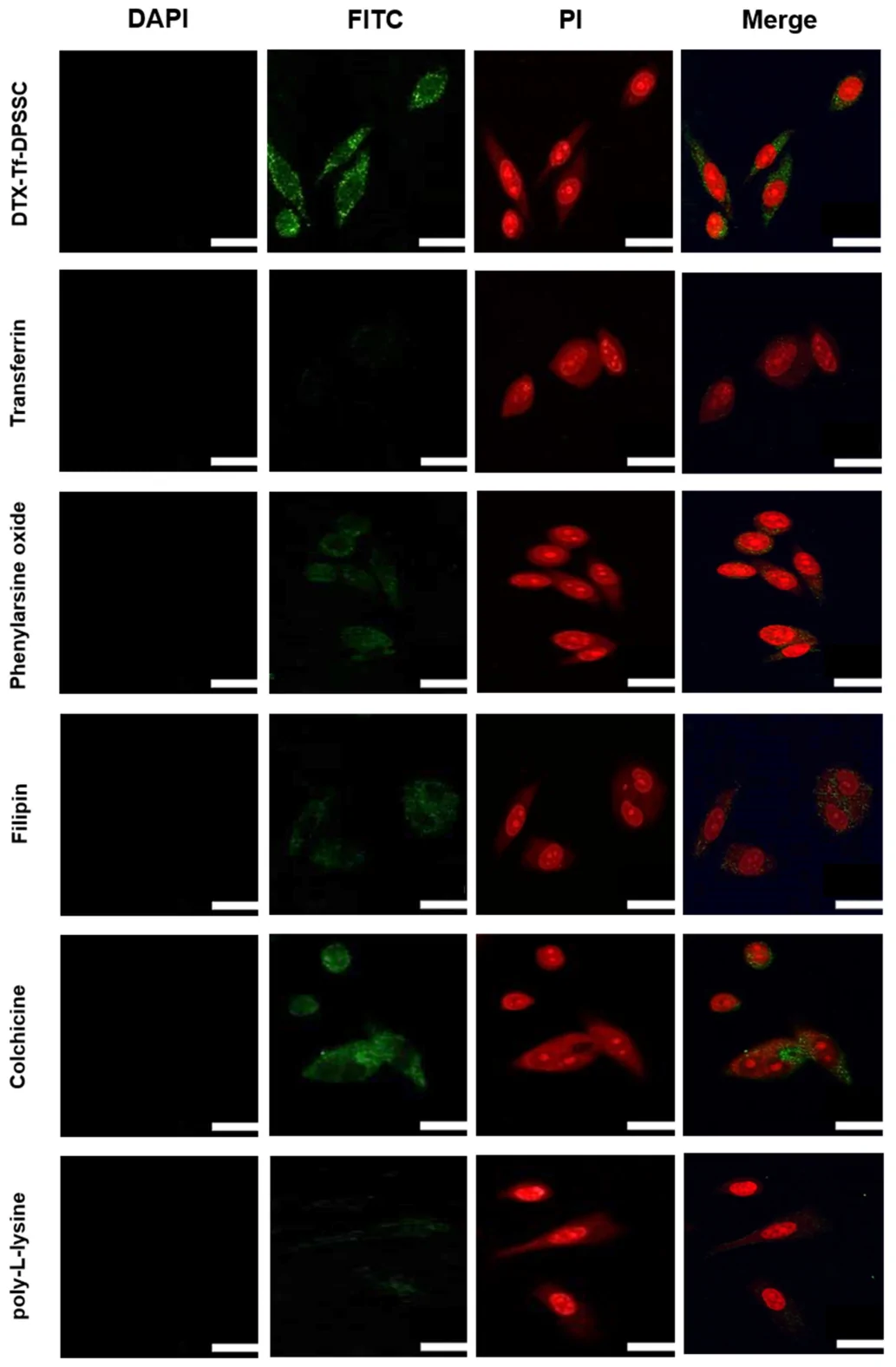
Confocal microscopy images showing the cellular uptake of fluorescein-labeled docetaxel entrapped in Tf-conjugated DPSSC in PC3-Luc cells following pre-treatment with free transferrin (Tf, 80 µM) or different uptake inhibitors: phenylarsine oxide, filipin, colchicine, and poly-L-lysine. Nuclei are shown in red following propidium iodide staining (excitation: 543 nm; emission: 597–647 nm), fluorescein-labeled docetaxel in green (excitation: 494 nm; emission: 517 nm), and camptothecin in blue (excitation: 405 nm; emission: 400–480 nm). Scale bar = 50 µm.

The cellular uptake of fluorescein-labeled docetaxel delivered by Tf-conjugated DPSSC appeared to involve multiple endocytic mechanisms. Confocal fluorescence was substantially reduced after pre-exposure to free Tf or endocytosis inhibitors. Free Tf and poly-L-lysine produced the strongest reductions, phenylarsine oxide and filipin had intermediate effects, and colchicine altered the uptake least. This pattern is consistent with a major contribution from specific receptor-associated uptake (Figure 10) [31].

**Figure 11:**
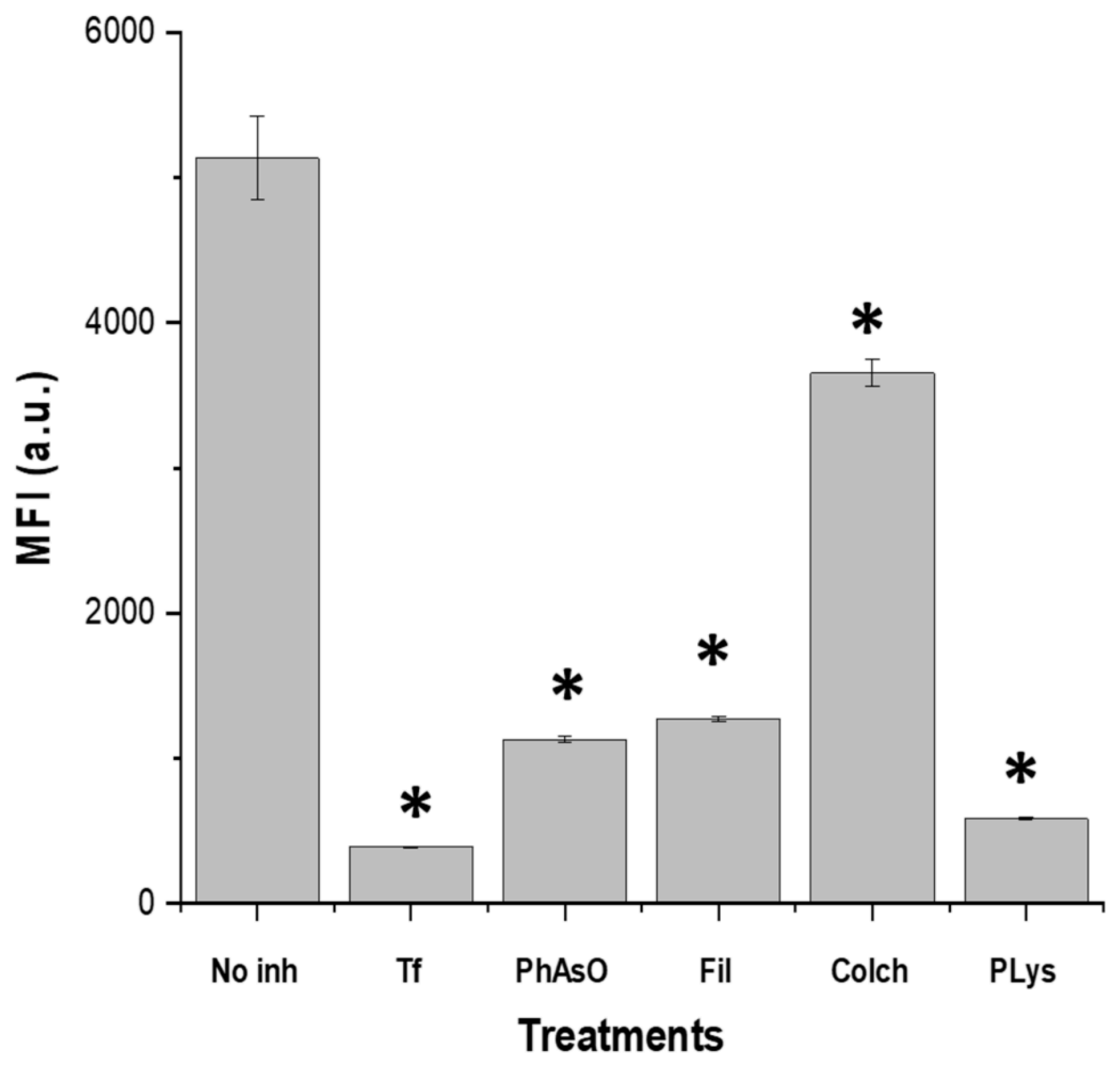
Flow cytometry analysis of the cellular uptake of fluorescein-labeled docetaxel entrapped in Tf-conjugated DPSSC in PC3-Luc cells following pre-treatment with free transferrin (Tf, 80 µM) or different uptake inhibitors: phenylarsine oxide (PhAsO), filipin (Fil.), colchicine (Colch), and poly-L-lysine (PLys) (*n* = 5). *: *p* < 0.05 compared with Tf-conjugated DPSSC entrapping fluorescein-labeled docetaxel.

Flow cytometry findings corroborated the confocal microscopy data. Pre-treatment of PC3-Luc cells with free transferrin (80 µM) significantly reduced the cellular uptake of fluorescein-labeled docetaxel delivered by Tf-conjugated DPSSC, producing a 13.12-fold decrease compared with cells that did not receive transferrin pre-treatment (391 ± 4 a.u. versus 5132 ± 288 a.u., respectively) (Figure 11). Pre-treatment with poly-L-lysine resulted in the next greatest inhibition of uptake, with an 8.77-fold decrease (585 ± 6 a.u.). Phenylarsine oxide and filipin also markedly reduced cellular uptake, by 4.53-fold and 4.03-fold, respectively (1132 ± 19 a.u. and 1272 ± 20 a.u.). In contrast, colchicine, used as a non-specific inhibitor, produced the weakest inhibitory effect, with only a 1.4-fold decrease in uptake (3655 ± 95 a.u.). These findings suggest that free transferrin competes with Tf-conjugated DPSSC for transferrin receptor binding, highlighting the central role of transferrin receptor-mediated endocytosis in the internalization of docetaxel-loaded Tf-conjugated DPSSC. They also indicate that additional endocytic pathways, including clathrin- and caveolae-mediated uptake, may contribute to cellular internalization.

### 3.9. Anti-Proliferative Efficacy

Transferrin conjugation of camptothecin-based dendrimersomes entrapping docetaxel significantly enhanced the anti-proliferative activity of both docetaxel and camptothecin in all three prostate cancer cell lines. Relative to a free docetaxel solution, Tf-conjugated DPSSC increased docetaxel efficacy by 3.47-fold in PC3-Luc cells, 3.72-fold in DU145 cells, and 3.88-fold in LNCaP cells. Likewise, compared with a free camptothecin solution, the anti-proliferative efficacy of camptothecin was enhanced by 4.56-fold, 5.07-fold, and 5.38-fold in PC3-Luc, DU145, and LNCaP cells, respectively (Table 1).

In PC3-Luc cells, Tf-conjugated DPSSC entrapping docetaxel showed the lowest IC_50_ valueof 11.72 ± 1.02 nM, indicating the highest anti-proliferative efficacy among the tested formulations. This was followed by control DPSSC loading docetaxel (26.28 ± 1.91 nM) and the combination of free docetaxel and camptothecin solutions (34.38 ± 2.97 nM). Higher IC_50_ values were recorded for free docetaxel solution (40.63 ± 1.40 nM) and free camptothecin solution (53.39 ± 4.58 nM) (Table 1).

A similar trend was observed in DU145 cells. Tf-conjugated DPSSC entrapping docetaxel again produced the lowest IC_50_ value (9.88 ± 1.22 nM), followed by control DPSSC loading docetaxel (20.04 ± 1.82 nM) and the combination of free docetaxel and camptothecin solutions (29.35 ± 2.57 nM). The corresponding IC_50_ values for free docetaxel and free camptothecin solutions were 36.75 ± 1.34 nM and 50.11 ± 4.74 nM, respectively (Table 1).

Among the three cell lines, LNCaP cells showed the greatest sensitivity to treatment, with the lowest IC_50_ values overall. In these cells, Tf-conjugated DPSSC entrapping docetaxel exhibited an IC_50_ of 7.88 ± 1.35 nM, compared with 13.66 ± 1.00 nM for control DPSSC loading docetaxel and 21.93 ± 1.17 nM for the combination of free docetaxel and camptothecin solutions. Free docetaxel and free camptothecin solutions showed IC_50_ values of 30.60 ± 1.52 nM and 42.39 ± 3.32 nM, respectively (Table 1). Together, these findings demonstrate that Tf-conjugated DPSSC provided the greatest anti-proliferative effect in all three prostate cancer cell lines, with the strongest response observed in LNCaP cells.

## 4. Discussion

Advanced prostate cancer, particularly metastatic castration-resistant prostate cancer, remains challenging to treat despite substantial therapeutic advances. Docetaxel continues to be a key first-line chemotherapeutic agent in advanced disease, and cabazitaxel is commonly used after docetaxel failure; however, durable benefit is frequently limited by intrinsic or acquired resistance, dose-limiting toxicity, and insufficient tumor selectivity. These limitations strongly support the development of tumor-targeted nanocarriers and rational drug combinations designed to improve intracellular drug delivery while reducing premature systemic exposure and off-target toxicity [5,6,34,35,36].

Within this context, the synergy observed here between docetaxel and camptothecin is particularly noteworthy. In PC3-Luc cells, the combination produced its strongest interaction at low nanomolar concentrations, with a minimum CI of 0.20 ± 0.01 and a corresponding growth inhibition of 88.10 ± 0.41%, and a second highly favorable combination yielded a CI of 0.32 ± 0.02 with 72.62 ± 1.56% growth inhibition. These data indicate that strong anti-proliferative activity can be achieved well below the concentrations typically associated with antagonism in the higher-dose combinations, which is mechanistically plausible because docetaxel disrupts mitosis through microtubule stabilization whereas camptothecin inhibits topoisomerase I and interferes with DNA replication and repair. This interpretation is consistent with the Chou–Talalay framework, in which CI values well below 1 indicate true synergy rather than simple additivity [10].

From a translational perspective, the choice of a near-optimal rather than the absolute best synergistic condition for downstream formulation is also reasonable, because practical manufacturability is an important consideration in nanomedicine development. This combination provided a CPT concentration that was more compatible with the preparation of the camptothecin-bearing dendrimer scaffold. The present synergy findings also compare favorably with earlier reports of docetaxel-based combinations in prostate cancer. Previous studies showed enhanced anti-cancer activity when docetaxel was combined with DLGAP5 suppression, AXL inhibition, mebendazole, doxorubicin co-delivery, capsaicin, or quercetin, confirming that docetaxel can benefit from rational partnering with agents that target complementary pathways [11,14,37,38,39,40,41]. However, these earlier strategies involved gene suppression, pathway inhibition, repurposed drugs, phytochemicals, or alternative co-delivery systems rather than a transferrin-targeted, dual stimulus-responsive dendrimersome simultaneously integrating docetaxel with a camptothecin-based scaffold. Although direct cross-study comparisons should be made cautiously because exposure time, cell model, assay conditions, and synergy metrics differ, the low CI values obtained here suggest that the docetaxel/camptothecin pairing is among the more promising synergistic combinations yet reported for prostate cancer cells. To our knowledge, this is the first report of such a combination being implemented in a transferrin-targeted camptothecin-based dendrimersome platform.

The design of the delivery system is also well supported by previous work on DAB dendrimers and transferrin-mediated targeting. Generation-3 DAB dendrimers were selected because earlier studies showed that this platform can provide efficient nucleic acid and drug delivery while maintaining a more favorable safety profile than more highly cationic dendrimer variants, and PEGylation has been shown to reduce toxicity while improving colloidal behavior and delivery performance [42]. In addition, heterobifunctional PEG chemistry and controlled bioconjugation strategies are well established for the generation of ligand-directed nanocarriers, and the use of such chemistry for transferrin attachment allows directional conjugation while avoiding uncontrolled crosslinking or polymerization [33]. Importantly, earlier work from the same broader research area established cholesterol-bearing and camptothecin-bearing PEGylated DAB dendrimersomes as redox-responsive platforms for drug and gene delivery, but did not combine transferrin targeting, physical docetaxel loading, and prostate cancer-directed evaluation within the same multifunctional construct [15,16,17]. The present study therefore extends those earlier dendrimersome concepts into a more advanced targeted combination-delivery strategy.

The physicochemical data further support the suitability of this formulation approach. Successful transferrin conjugation, preservation of self-assembly, and the maintenance of a CAC comparable to that of the parent camptothecin-bearing dendrimersomes indicate that ligand functionalization did not compromise vesicle formation. This is an important point, because active targeting ligands can sometimes destabilize self-assembled systems or alter their supramolecular organization. The high entrapment efficiency obtained for docetaxel also compares favorably with several previously reported docetaxel nanocarriers, including folate-conjugated TPGS micelles, poly(ε-caprolactone)/Pluronic nanoparticles, and transferrin-functionalized liposomes [25,43,44,45,46]. By contrast, the lower apparent camptothecin loading is not unexpected, because camptothecin in this system is not merely co-encapsulated as a passive payload but is incorporated as a conjugated structural component of the dendrimersome scaffold itself. The present platform should therefore be viewed as a hybrid nanomedicine in which camptothecin contributes both therapeutic function and nanostructural organization, while docetaxel is subsequently entrapped within the vesicular system.

The release studies are also consistent with the intended design of the system. Docetaxel showed an initial burst release followed by a more sustained profile, whereas the cumulative release of both drugs increased under acidic conditions relative to pH 7.4. This behavior is desirable for intravenous delivery because it suggests reduced premature release under physiological conditions and greater liberation of the payload in the more acidic extracellular and intracellular environments associated with tumors. In parallel, camptothecin release increased markedly at high glutathione concentrations, consistent with reductive cleavage of the disulfide linkage used in the camptothecin-bearing scaffold. More generally, controlled release from polymeric nanocarriers is known to depend on diffusion, water uptake, matrix relaxation, and degradation processes, whereas redox-responsive systems exploit the higher intracellular glutathione concentrations found in many tumor cells to trigger selective drug liberation [47,48]. The present results are also in line with previous reports on camptothecin-bearing DAB dendrimersomes, in which redox-triggered release was retained under reducing conditions [16]. The translational relevance of reduction-sensitive docetaxel delivery is further supported by a recent PSMA-targeted, disulfide-linked PEG–docetaxel nanoprodrug that suppressed prostate cancer progression [49]. Together, these findings indicate that Tf conjugation preserved the pH- and redox-responsive behavior of the parent platform while adding an active targeting function. The selected pH and GSH conditions represent physiologically informed, compartment-specific environments that may be encountered sequentially by the formulation. Following systemic administration, the dendrimersomes would initially be exposed to physiological pH and low extracellular GSH concentrations. Following tumor accumulation and receptor-mediated internalization, they may encounter mildly acidic tumor interstitium, progressive endosomal acidification, and, after access to the cytosol, a substantially more reduced millimolar GSH environment. The 50 mM GSH condition should be regarded as an accelerated, strongly reducing challenge rather than a direct physiological simulation. Furthermore, these simplified in vitro release conditions cannot fully reproduce the spatial heterogeneity, protein interactions, cellular trafficking, clearance and dynamic pH/redox gradients present in vivo. Consequently, the results demonstrate the potential pH- and redox-responsive behavior of the formulation, but confirmation will require biodistribution and therapeutic studies in tumor-bearing models.

One of the clearest functional advantages of the present system was the marked enhancement in cellular uptake. Both confocal microscopy and flow cytometry showed substantially greater uptake of docetaxel when it was delivered by Tf-conjugated DPSSC than by non-targeted dendrimersomes or free drugs. The inhibition studies strongly support a dominant role for transferrin receptor-mediated endocytosis, with additional contributions from clathrin- and caveolae-associated pathways. This interpretation is biologically plausible because the transferrin receptor is frequently overexpressed in proliferating cancer cells, including prostate cancer cells, owing to their high iron requirements [19,21,22,27,30,50]. The stronger uptake observed in LNCaP cells than in DU145 and PC3-Luc cells is also consistent with previous reports suggesting differential TfR1 expression across prostate cancer cell lines. Similar uptake-enhancing effects have been reported for transferrin-modified nanocarriers in several tumor models, including paclitaxel-loaded transferrin-targeted nanoparticles, transferrin-functionalized liposomes for docetaxel delivery, transferrin-bearing zein-based hybrid nanoparticles, and other TfR-directed delivery systems [23,25,26,28,29,30,51,52]. However, the magnitude of the uptake enhancement observed here appears particularly strong, especially considering that it was achieved in a multifunctional dual-drug platform rather than in a simpler single-drug carrier.

Nevertheless, the targeting advantage conferred by transferrin should be interpreted in light of the non-exclusive expression of TfR1 in malignant tissues. Although TfR1 is frequently upregulated in cancer cells because of their increased iron requirements and proliferative activity, it is also the principal receptor responsible for transferrin-bound iron uptake in many normal cell types and is particularly important in developing erythroid cells [27,29,30,53]. TfR1-mediated iron uptake is essential for hemoglobin synthesis and normal erythropoiesis, indicating that erythroid precursors in the bone marrow may represent particularly relevant potential off-target cells [30,53]. Consequently, transferrin conjugation should be regarded as providing preferential rather than absolute tumor targeting.

The improved intracellular delivery translated into a marked enhancement in anti-proliferative efficacy. Tf-conjugated DPSSC consistently produced the lowest IC_50_ values among all formulations in PC3-Luc, DU145, and LNCaP cells, confirming that the benefits of active targeting and controlled intracellular release were functionally meaningful. The strongest anti-proliferative effect was observed in LNCaP cells, which is in keeping with the higher uptake seen in this cell line and with the broader literature, suggesting stronger transferrin receptor expression in LNCaP than in DU145 or PC3-derived cells [21,22]. Previous studies have shown that targeted nanocarriers can improve the cytotoxicity of docetaxel or other anti-cancer agents in prostate cancer models, including transferrin-conjugated liposomes, transferrin-bearing dendriplexes, transferrin-bearing zein-based hybrid nanoparticles, PSMA-targeted dual-drug nanoparticles, PEGylated solid lipid nanoparticles, and mesoporous silica nanomedicines [14,24,25,26,45,46]. Nevertheless, most earlier systems focused on either single-drug delivery or one dominant functional feature. The present study is more distinctive because it integrates a synergistic docetaxel–camptothecin combination, transferrin receptor targeting, and dual pH/redox responsiveness within a single self-assembled dendrimersome construct.

These findings indicate that the present Tf-conjugated DPSSC system is more than an incremental reformulation of docetaxel. Rather, it represents a multifunctional nanocarrier in which camptothecin serves both as a therapeutic component and as a structural element of a redox-responsive dendrimersome; docetaxel is efficiently entrapped as a second cytotoxic payload; transferrin promotes receptor-mediated uptake; and acidic/reductive tumor-associated conditions favor drug release after internalization. This multimodal design is, to the best of our knowledge, the principal novelty of the work. It provides a strong rationale for future evaluation in more advanced prostate cancer models, including 3D systems and in vivo tumor-bearing models, where the full therapeutic benefit of active targeting and tumor-responsive release may become even more apparent [14,16,17,26,45,46,49].

## 5. Conclusions

This work reports, for the first time, a transferrin-conjugated dendrimersome platform in which camptothecin is covalently incorporated into the dendrimer scaffold through a disulfide linkage, and docetaxel is physically entrapped within the self-assembled vesicles, for targeted prostate cancer therapy. This multifunctional delivery system successfully integrates synergistic dual-drug treatment, tumor-targeting capability, and stimulus-responsive release within a single nanocarrier. Tf-conjugated DPSSC demonstrated high docetaxel entrapment efficiency, enhanced cellular uptake predominantly through transferrin receptor-mediated endocytosis, controlled pH- and redox-responsive release of both drugs, and superior anti-proliferative efficacy in three prostate cancer cell lines compared with non-targeted formulations and free-drug solutions. These findings identify Tf-conjugated DPSSC as a promising nanomedicine platform for the co-delivery of covalently conjugated camptothecin and physically entrapped docetaxel and support its further evaluation as a targeted therapeutic strategy for advanced prostate cancer.

## Figures and Tables

**Table 1:** Anti-proliferative efficacy of Tf-conjugated DPSSC entrapping docetaxel, control DPSSC loading docetaxel, a combination of free docetaxel and camptothecin solutions, and the individual free docetaxel and camptothecin solutions in PC3-Luc, DU145, and LNCaP prostate cancer cell lines. Results are expressed as mean ± S.E.M. (*n* = 15).

	IC_50_ (nM) (Mean ± S.E.M.)				
	Formulations				
Cell Line	TF-DPSSC-DTX	DPSSC-DTX	CPT-DTX	DTX	CPT
PC3-Luc	11.72 ± 1.02	26.28 ± 1.91	34.38 ± 2.97	40.63 ± 1.40	53.39 ± 4.58
DU145	9.88 ± 1.22	20.04 ± 1.82	29.35 ± 2.57	36.75 ± 1.34	50.11 ± 4.74
LNCaP	7.88 ± 1.35	13.66 ± 1.00	21.93 ± 1.17	30.60 ± 1.52	42.39 ± 3.32

## Data Availability

The data that support the findings of this study are available from the corresponding author upon request.

## Abbreviations

The following abbreviations are used in this manuscript:

ANOVA	One-way analysis of variance
A.u.	Arbitrary unit
CAC	Critical aggregation concentration
CDCl_3_	Deuterated chloroform
CI	Combination index
Colch	Colchicine
CPT	Camptothecin
DAB	Generation-3 diaminobutyric polypropylenimine dendrimer
DLGAP5	Discs large-associated protein 5
DMAP	4-dimethylaminopyridine
DMSO	Dimethyl sulfoxide
DMSO-d_6_	Deuterated dimethyl sulfoxide
D_2_O	Deuterium oxide
DPSSC	Disulfide-linked camptothecin-bearing PEGylated DAB dendrimer
DTX	Docetaxel
EDC	N-(3-dimethylaminopropyl)-N′-ethylcarbodiimide hydrochloride
Fil	Filipin
GMBS	N-γ-maleimidobutyryl-oxysuccinimide ester
GSH	Glutathione
HEPES	4-(2-hydroxyethyl)-1-piperazineethanesulfonic acid
IC_50_	Half-maximal inhibitory concentration
MEM	Minimum essential Eagle’s medium
MFI	Mean fluorescence intensity
MTT	3-(4,5-dimethylthiazol-2-yl)-2,5-diphenyltetrazolium bromide
MWCO	Molecular weight cut-off
NMR	Nuclear magnetic resonance
OPSS-PEG-SCM	Orthopyridyl disulfide polyethylene glycol succinimidyl carboxymethyl ester
PBS	Phosphate-buffered saline
PEG	Polyethylene glycol
PhAsO	Phenylarsine oxide
PLys	Poly-L-lysine
RPMI	Roswell Park Memorial Institute
S.E.M.	Standard error of the mean
TEM	Transmission electron microscopy
Tf	Transferrin
TfR1	Transferrin receptor 1
TPGS	Tocopheryl polyethylene glycol 1000 succinate
